# SQSTM1/p62 Orchestrates Skin Aging via USP7 Degradation

**DOI:** 10.1111/acel.70078

**Published:** 2025-05-08

**Authors:** Liu Chen, Xiaoping Wang, Yuchen Wang, Qingxin Yao, Yunyao Liu, Yongcheng Zhu, He Huang, Hedan Yang, Yin Yang, Yuan He, Lei Qiang

**Affiliations:** ^1^ State Key Laboratory of Natural Medicines, School of Basic Medicine and Clinical Pharmacy China Pharmaceutical University Nanjing China; ^2^ Global Platform One Vision WuXi AppTec Shanghai China; ^3^ Jiangsu Key Laboratory of Molecular Biology for Skin Diseases and STIs, Institute of Dermatology Chinese Academy of Medical Sciences & Peking Union Medical College Nanjing China; ^4^ Shanghai Institute of Materia Medica Chinese Academy of Sciences Shanghai China; ^5^ Hospital of Dermatology Chinese Academy of Medical Sciences and Peking Union Medical College Nanjing China

**Keywords:** binding interaction, skin aging, SQSTM1/p62, USP7

## Abstract

Skin aging is a complex process driven by intrinsic genetic factors and extrinsic environmental influences. In this study, sequestosome1 (SQSTM1/p62) was identified as a key regulator of senescence, the senescence‐associated secretory phenotype (SASP), and skin aging. Notably, p62 expression is reduced in senescent cells and aging skin of both humans and mice. The depletion of p62 in the epidermis was found to be positively associated with accelerated aging and the initiation of SASP. Mechanistically, p62 inhibits the accumulation of ubiquitin‐specific protease 7 (USP7) during senescence induction by orchestrating its degradation through specific binding interactions. In particular, the Tyr‐67 residue within the PB1 domain or Gln‐418 within the UBA domain of p62 forms a hydrogen bond with Ala‐993 in the Ubl5 domain of USP7. Mutations in either Tyr‐67 or Gln‐418 of p62, or Ala‐993 of USP7, resulted in the induction of cellular senescence, highlighting the critical role of these molecular interactions in the regulation of aging processes.

## Graphical abstract

p62 exhibits diminished expression in aging skin and senescent cells. p62 serves to inhibit the accumulation of USP7 during senescence induction. The Tyr‐67 residue within the PB1 domain or Gln‐418 within the UBA domain of p62 forms a hydrogen bond with Ala‐993 of the Ubl5 domain of USP7.

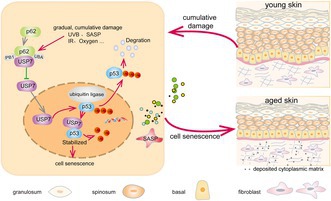

**Abbreviations**
Co‐IP: co‐immunoprecipitation
DLB: dementia with Lewy bodies
GO: gene ontology
HAUSP: herpes virus‐associated protease
KEGG: kyoto encyclopedia of genes and genomes
KIR: keap1‐interacting region
LIR: LC3‐interacting region
MDM2: mouse double minute 2 homolog
MSA: multiple‐system atrophy
p62‐KD: p62‐knockdown
PB1: phox1 and bem1p
PD: parkinson disease
PDB: paget disease of bone
SASP: senescence‐associated secretory phenotype
SA‐β‐gal: senescence‐associated β‐galactosidase
SQSTM1 or p62: sequestosome1
TBS: tumor necrosis factor receptor‐associated factor 6 (TRAF6)‐binding (TB) motif
TEWL: transepidermal water loss
TRAF: tumor necrosis factor receptor‐associated factors
UBA: ubiquitin associated
Ubl: ubiquitin like
UPS: ubiquitin–proteasome system
USP7: ubiquitin‐specific protease 7
UVB: ultraviolet radiation B
UVR: ultraviolet radiation
ZZ: zinc finger

## 1 Introduction

Skin, the largest organ of the body (Gallo 2017), serves as a critical physical barrier against ultraviolet radiation, air pollution, and pathogens (Celebi Sozener et al. 2022). With aging, the skin becomes increasingly susceptible to diseases and malignancies (Kammeyer and Luiten 2015), while also reflecting overall health status, mortality risk, and longevity (Franco et al. 2022). The epidermal layer, functioning as the foremost defensive barrier, not only protects against external microbial agents and environmental stimuli, but also plays essential roles in absorption, immunological response, and oxidative processes (Gallo 2017). In the dermis, fibroblasts regulate collagen expression and maintain skin integrity (Bahudhanapati et al. 2024; Gu et al. 2020). However, senescent fibroblasts contribute to dermal thinning, increased wrinkle formation, and skin sagging (Zhang et al. 2024). Keratinocytes also play a pivotal role in shaping the senescent skin microenvironment, including the maintenance of the dermal–epidermal junction (Bahudhanapati et al. 2024) and the secretion of senescence‐associated secretory phenotype (SASP) factors (Zhang et al. 2022). Notably, senescent keratinocytes exhibit enrichment of SASP components, including proinflammatory cytokines and proteases, with upregulated genes, IL‐1β as a key mediator of chronic inflammation in aging tissues (Xu et al. 2024). The consequent decline in cellular and tissue regenerative potential is implicated in the progression of skin aging. However, the regulatory networks governing skin aging in response to epidermal senescence remain incompletely understood, highlighting the need for further investigation.

p53 is central for genetic stability and cell cycle regulation, initiating key processes such as cell cycle arrest, DNA repair, and senescence (Lopez‐Otin et al. 2023; Wu and Prives 2018). Its inhibition allows previously arrested cells with low p16 levels to re‐enter the cell cycle, suggesting that senescence is reversible under certain conditions (Vaddavalli and Schumacher 2022). Studies have shown that inhibiting active p53 has a beneficial role in the aging process of the skin (Kim et al. 2014; Lauri et al. 2014). As a known target of ubiquitin‐specific protease 7 (USP7), reactivating p53 is considered a potential strategy against premature senescence (Zeng et al. 2022).

USP7, also known as herpesvirus‐associated protease (HAUSP), regulates intracellular protein homeostasis through selective substrate degradation (Pozhidaeva and Bezsonova 2019). It plays a crucial role in cell cycle control, senescence, and cancer by interacting with diverse target proteins (Cui et al. 2020; Granieri et al. 2022; Nininahazwe et al. 2021). USP7 contains three functional domains, including the tumor necrosis factor receptor‐associated factors (TRAF) domain, which binds proteins like MDM2 and p53, the catalytic domain mediating deubiquitination, and the C‐terminal tandem ubiquitin‐like (Ubl) domain where Ubl4 and Ubl5 are sufficient for activation (Rouge et al. 2016; Wang et al. 2019; Zhu et al. 2007). Additionally, the USP7/p300 complex enhances p53 expression and activates p21, leading to cell cycle arrest and premature senescence of endothelial progenitor cells (Zeng et al. 2022). USP7 depletion reduces cellular lifespan and stress resistance, while USP7 inhibitors have been shown to eliminate senescent cells and mitigate doxorubicin‐induced SASP in mice (Cui et al. 2020; He et al. 2020). Although previous studies have highlighted a potential role for active USP7 in cellular senescence, its precise mechanisms, particularly in the regulation of skin aging via a p53‐independent pathway, remain to be fully elucidated.

Sequestosome1 (SQSTM1 or p62), hereafter p62, an autophagy receptor, has been associated with aging and age‐related diseases, including neurodegeneration, infections, cancer, and oxidative stress‐related conditions (Kaushik et al. 2021; Kocak et al. 2022; Ma et al. 2019; Tao et al. 2020). p62 deficiency is associated with a shorter lifespan, elevated oxidative stress (Kwon et al. 2012), synaptic deficiencies, and memory impairment (Ramesh Babu et al. 2008). By interacting with GATA4, p62 promotes selective autophagic degradation, inhibiting cellular senescence (Xiong et al. 2022). As a scaffold protein, p62 regulates the formation and clearance of ubiquitinated aggregates through its interaction domains and ubiquitin‐binding regions, thereby modulating specific signaling pathways (Lee et al. 2022). In Drosophila, mutants lacking the Phox1 and Bem1p (PB1) or ubiquitin‐associated (UBA) domains exhibit shortened lifespans, emphasizing the critical role of these domains (de Castro et al. 2013). Additionally, JunB suppresses p62 expression in keratinocytes, linking it to psoriasis‐related inflammation (Sukseree et al. 2021). However, the precise mechanisms through which p62 regulates keratinocytes in skin aging are unknown.

In this study, we investigate the function of p62 and potential mechanisms in skin aging and cellular senescence. We identified p62 as a negative regulator in skin aging and senescent keratinocytes. Subsequently, we demonstrated its inhibition of USP7 accumulation and USP7‐mediated p53/p21 pathway activation. Moreover, mutations in p62 (Tyr‐67, Gln‐418) or USP7 (Ala‐993) induced senescence. cellular senescence. Importantly, this study provides the first time, to our knowledge, that p62 plays a critical role and regulates specific mechanisms in skin aging and cellular senescence.

## 2 Results

### 2.1 p62 Expression is Reduced in Aging Skin

**FIGURE 1:**
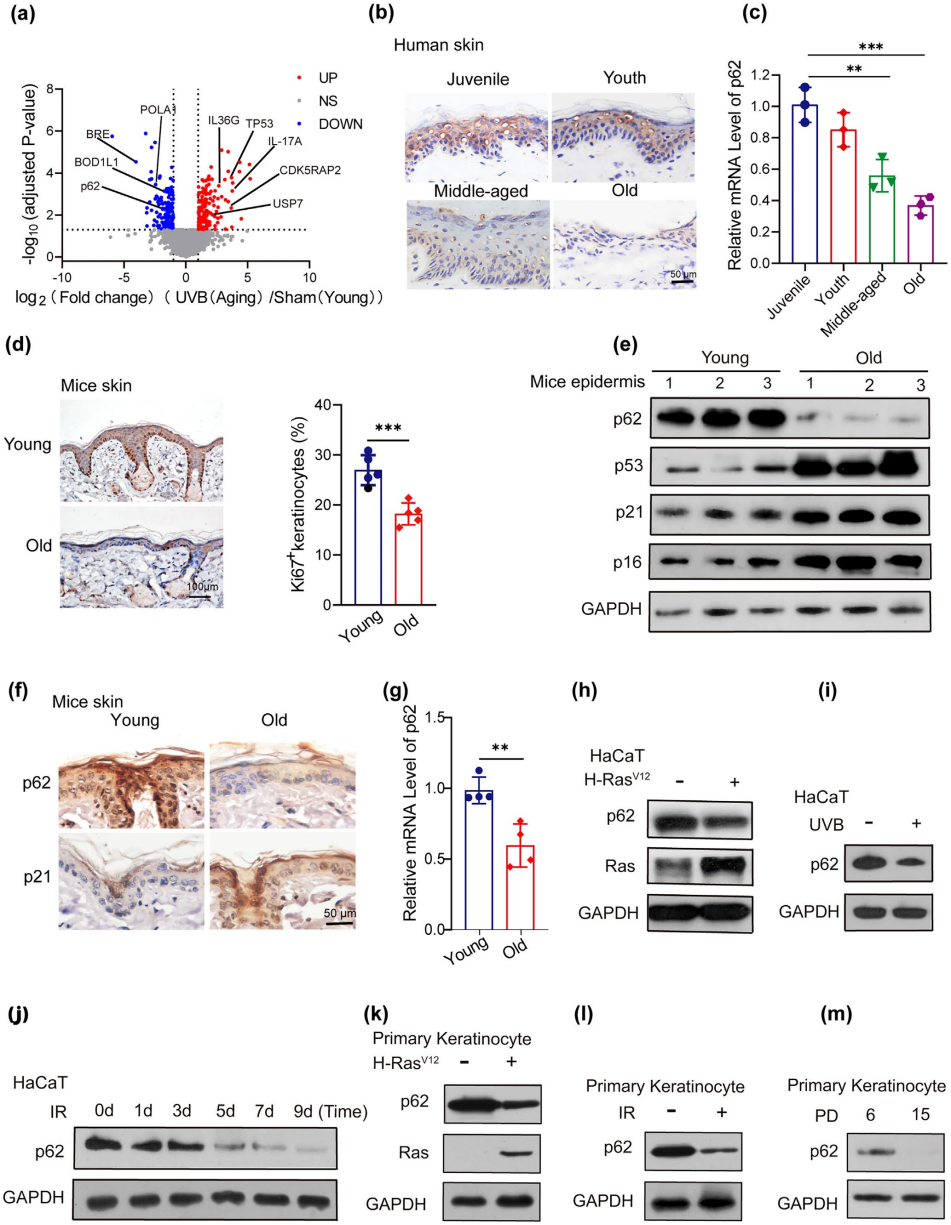
p62 is reduced in senescent cells and aging skin. (a) Quantitative proteomics analysis of significantly up‐ (red) or downregulated (blue) proteins of more than 4000 proteins in normal and UVB‐induced senescent human keratinocyte cells (*p* < 0.05, *n* = 3). (b) Immunohistochemical analysis of p62 was performed in normal human skin epidermis of juvenile (13 ~ 17 years old), youth (18 ~ 45 years old), middle‐aged (46 ~ 69 years old), and old (> 69 years old) individuals. Scale bar: 50 μm. (c) Relative abundance of p62 mRNA was expressed with different age groups in human skin epidermis. (d) Representative images of Ki67 staining were detected in young (2‐month‐old) and old (20‐month‐old) skin (left), and quantitative analysis of Ki67^+^ cells in the epidermis was performed (right). Scale bar: 100 μm. (e) Western blotting showed expression of p62 and proteins of aging‐related markers (p53, p21, and p16) in skin epidermis from young and old SKH1 mice skin epidermis. (f) Representative immunohistochemical staining of p62 and p21 in skin epidermis from young and old mice. Scale bar: 50 μm. (g) RT‐PCR quantified relative p62 mRNA expression of skin epidermis from young and old mice. (h‐j) Western blotting assay indicated expression of p62 in keratinocytes during oncogene (H‐Ras^V12^)‐, UVB‐, and IR‐induced senescence. (k‐m) Western blotting assay showed expression of p62 in murine primary keratinocytes during oncogene (H‐Ras^V12^)‐, IR‐induced senescence, or replicative senescence. Data show mean ± SEM. Comparisons by two‐way ANOVA for c. Comparisons by unpaired t test for d and g. ***p < 0.001, **p < 0.01.

Numerous signaling pathways, including p53‐mediated cell cycle arrest and GATA4‐regulated SASP, have been implicated in the regulation of cellular senescence (Xiong et al. 2022). Ultraviolet B radiation (UVB) is a key factor that induces and accelerates skin aging. To identify novel predominant proteins involved in UVB‐accelerated skin aging, differently‐ expressed proteins were analyzed in normal and senescent human keratinocytes irradiated with 5 mJ/m^2^ UVB for five exposures (Figure S1a). Results from quantitative proteomics analysis revealed several signature proteins (e.g., IL36, TP53, and USP7) were significantly upregulated, while proteins (e.g., p62 and POLA1) were significantly downregulated (Figure 1a). Meanwhile, the Kyoto Encyclopedia of Genes and Genomes (KEGG) and Gene Ontology (GO) analysis indicated that DNA replication and lysosome activity played pivotal roles in these settings. The upregulated genes were predominantly enriched in pathways related to the cell cycle (Figure S1b,c). To assess alterations in p62 expression during human skin aging, we detected its gene and protein expression levels in the epidermis of different age groups (juvenile, youth, middle aged, and old). Immunohistochemistry (IHC) and reverse transcription quantitative real‐time polymerase chain reaction (RT‐qPCR) showed a significant reduction in p62 expression in the epidermis of older individuals compared to younger donors (Figure 1b,c). Similarly, aged murine skin exhibited reduced water retention in the stratum corneum (Figure S1d,e). Histological analysis revealed epidermal thinning and progressive collagen fiber degradation in aged mouse skin compared to young mice (Figure S1f,g). Moreover, aged mouse skin displayed fewer proliferative cells and an increased number of senescent cells relative to young skin (Figures 1d and S1h). Aligned with these observations, the aged murine epidermis exhibited reduced p62 expression and increased aging markers (p53, p21, and p16) compared to the young epidermis (Figure 1e–g). SASP can promote further cellular senescence and extracellular matrix deterioration (Ghosh and Capell 2016; Narzt et al. 2021). Chronic SASP may limit clearance and propagate the senescence and aging phenotype (Coppe et al. 2010). A significant upregulation in the expression and secretion of multiple SASP genes, along with inflammatory and immune‐modulatory cytokines, was observed in senescent cells (Figure S1i,j). Additionally, p62 expression was significantly decreased upon oncogene‐, UVB‐, and IR‐induced senescence of keratinocytes (Figure 1h–j) and oncogene‐, IR‐induced senescence, or replicative senescence of primary keratinocytes (Figure 1k–m). These findings indicate that p62 expression is diminished in both aged human and mouse skin, as well as in senescent cells, underscoring its critical role in the regulation of skin aging.

### 2.2 Epidermal p62 Deficiency Accelerates Skin Aging in Mice

**FIGURE 2:**
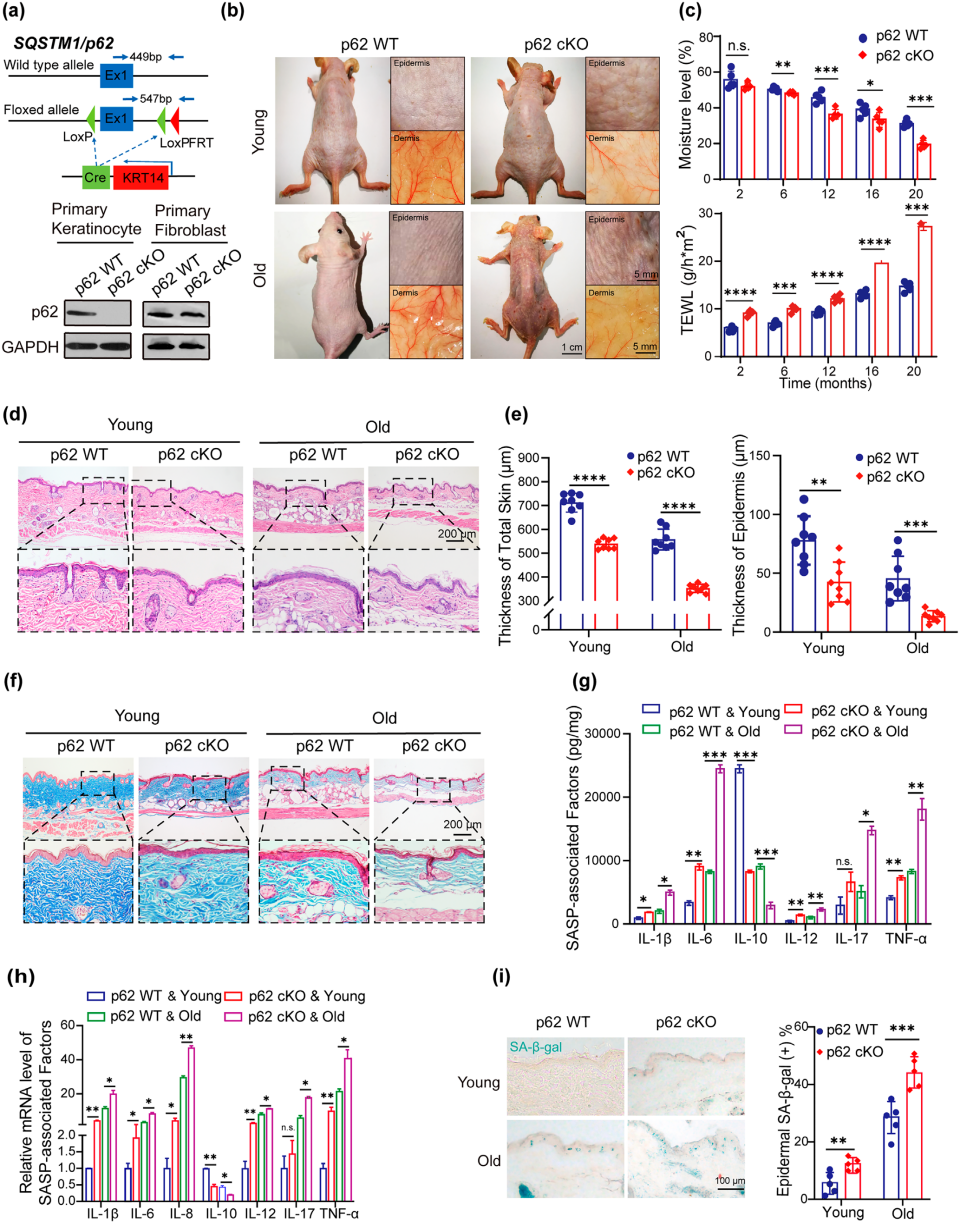
Epidermal p62 deficiency accelerates mice's skin aging. (a) Construction (top) and identification (bottom) of epidermal p62 deficiency SKH1 mice: *P62* WT and *p62* cKO mice. (b) Skin symptoms were assessed in *p62* WT and *p62* cKO SKH1 mice at 2 months old (young) and 20 months old (old). Scale bars: 1 cm or 5 mm. (c) Moisture (top) and TEWL (bottom) measurements detected percutaneous water loss of the stratum corneum of epidermal p62 deficiency mice at indicated ages. (d) HE stained images of mouse skin were obtained. Scale bar: 200 μm. (e) Quantitative analysis of skin epidermal and dermal thickness. (f) Masson trichrome staining detected the collagen remodeling. Scale bar: 200 μm. (g) ELISA assay measured the secretion of senescence‐associated secretory phenotype (SASP) factors. (*n* = 5). (h) The expression of SASP genes was quantified by RT‐qPCR. (i) Representative images of SA‐β‐gal staining were detected (left), and quantitative analysis of epidermal SA‐β‐gal‐positive cells was performed (right). Scale bar: 100 μm. Data show mean ± SEM. Comparisons by unpaired *t* test for c, e and i. Comparisons by two‐way ANOVA for g and h. ****p < 0.0001, ***p < 0.001, **p < 0.01, *p < 0.05, n.s., not significant.

To further investigate the role of p62 in skin aging, a p62 conditional knockout hairless mice model in keratinocytes with identified route and characterization, namely, *p62* ^*f/f*^; K14 Cre (*p62* cKO) mice and its control *p62* ^*f/f*^ mice (*p62* WT) were characterized and genotyped (Figures 2a and S2a). The successful deletion of p62 in keratinocytes was confirmed by genotyping and analysis of primary keratinocytes, excluding mice with unsuccessful deletions (Figures 2a and S2b). *p62* cKO mice showed skin aging‐like symptoms such as roughness, stiffness, lack of elasticity, deepening, and increased wrinkles (Figure 2b). Measured moisture level and transepidermal water loss (TEWL) were measured at 2, 6, 12, 16, and 20 months old. Epidermal p62 deficiency mice significantly reduced moisture levels and increased TEWL compared to WT mice (Figure 2c). Additionally, epidermal and skin thickness were reduced in young p62 cKO mice compared to *p62* WT, with the difference becoming more pronounced with aging (Figure 2d,e). Loss of collagen fibers in mouse dermis was detected in epidermal p62 deficiency mice skin (Figure 2f). Furthermore, inflammatory and immune‐modulatory cytokines secreted by senescent cells enhanced senescence arrest and altered the inflammatory microenvironment in young *p62* cKO compared to *p62* WT, with these effects becoming more pronounced in older mice (Figure 2g,h). Notably, epidermal p62 deficiency led to senescence, as indicated by reduced proliferative capacity and an elevated number of SA‐β‐gal‐positive cells (Figure 2i; Figure S2c,d). Together, these data demonstrated that epidermal p62 deficiency accelerates skin aging and contributes to a pro‐inflammatory microenvironment.

### 2.3 p62 Is a Potential Modulator of Keratinocyte Senescence

**FIGURE 3:**
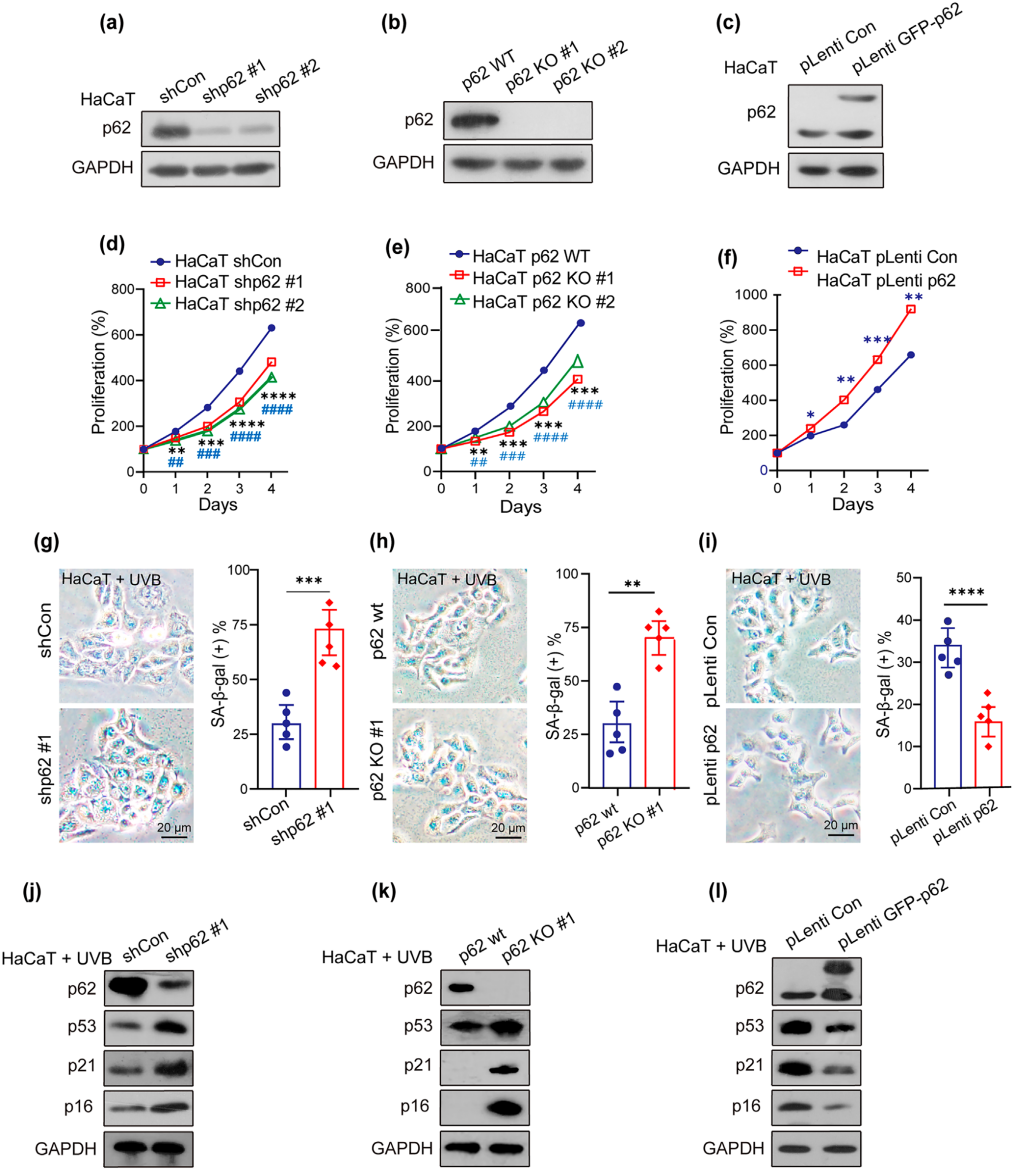
p62 regulates cellular senescence. (a, b and c) Western blotting showed the expression of p62 in shCon and shp62 (p62 wt, p62 KO/pLenti Con, and pLenti GFP‐p62) HaCaT cells. (d, e, and f) CCK8 assay showed the cell viability in shCon and shp62 (p62 wt, p62 KO/pLenti Con, and pLenti GFP‐p62) HaCaT cells. (g, h and i) Representative images of SA‐β‐gal staining were shown in shCon and shp62 (p62 wt, p62 KO/pLenti Con, and pLenti GFP‐p62) HaCaT cells with 5 mJ/m^2^ UVB treatment (UVB) (left) and the quantification of SA‐β‐gal‐positive cells were performed (right). Scale bar: 20 μm. (j, k and l) Western blotting tested the expression of p62 and aging‐related biomarkers (p53, p21, and p16) in shCon and shp62 (p62 wt, p62 KO/pLenti Con, and pLenti GFP‐p62) HaCaT cells with UVB treatment. Data show mean ± SEM. Comparisons by two‐way ANOVA for d‐f. Comparisons by unpaired  t Comparisons by two‐way ANOVA for test for g‐i. ****p < 0.0001, ***p < 0.001, **p < 0.01, ^# # # #^ *p* < 0.0001, ^# # #^ *p* < 0.001, ^# #^ *p* < 0.01.

To investigate whether p62 regulates cellular senescence in keratinocytes, several cell models, including IMR90 and HaCaT, were applied to verify our hypothesis. IMR90 cells are commonly used for aging research due to their characteristic features. Firstly, p62‐knockdown (p62‐KD) and p62‐overexpressing IMR90 cell pairs were established (IMR90.shp62 vs. IMR90.shCon and IMR90.pLenti p62 vs. IMR90.pLenti Con) (Figure S3a–d). SA‐β‐gal staining confirmed that p62‐KD significantly increased cellular senescence in IMR‐90 fibroblasts compared to the controls (Figure S3e), while p62 overexpression reduced senescence (Figure S3f). Subsequently, this phenomenon was verified in human‐immortalized keratinocytes. Repeated excessive exposure to ultraviolet radiation (UVR), particularly UVB, induces skin alterations resembling chronological aging. UVB accelerates and induces premature senescence in normal human epidermal keratinocytes (Ge et al. 2024). Similar to the IMR90 cell pair, p62‐KD, p62 knockout (p62‐KO) using the CRISPR‐Cas system, and p62‐overexpressing HaCaT cell pairs were established and validated (Figure 3a–c). Cell proliferation assays from all these cell pairs demonstrated that p62 reduction or depletion significantly decreased the capacity of HaCaT cell growth (Figure 3d,e) and that overexpression of p62 could salvage this situation (Figure 3f). Results from SA‐β‐gal staining of UVB‐treated p62‐KD or p62‐KO HaCaT cell pairs confirmed that p62 reduction or depletion promoted cellular senescence (Figure 3g,h), whereas p62 overexpression exerted a protective effect, reducing senescence (Figure 3i). Furthermore, aging‐related biomarkers (p53, p21, and p16) were significantly upregulated in UVB‐treated p62‐KD and p62‐KO HaCaT cells compared to UVB‐treated control cells (Figure 3j,k). In contrast, p62 overexpression in HaCaT cells resulted in a lower expression of p53, p21, and p16. (Figure 3l). Collectively, these observations substantiate the role of p62 as a negative regulator of senescence.

### 2.4 p62 Inhibits the Abundance of USP7 and USP7‐Mediated p53/p21 Pathway in Response to Senescence Induction

Given that p62 acts as a negative regulator of senescence, it is imperative to explore the mechanisms underlying its regulation of this process. Activation of either the p53/p21 or p16/pRB pathway leads to cell cycle arrest (Wu and Prives 2018). To determine whether p62 regulates cellular senescence in a p53/p21‐dependent manner, we established a stable p53 knockdown in shp62 keratinocytes (Figure S4a). Notably, p53 reduction effectively abolished the inhibitory effects of p62 on senescence (Figure S4b–d) and SASP factors (Figure S4e), highlighting the essential role of the p53 pathway exists in p62‐mediated senescence regulation.

**FIGURE 4:**
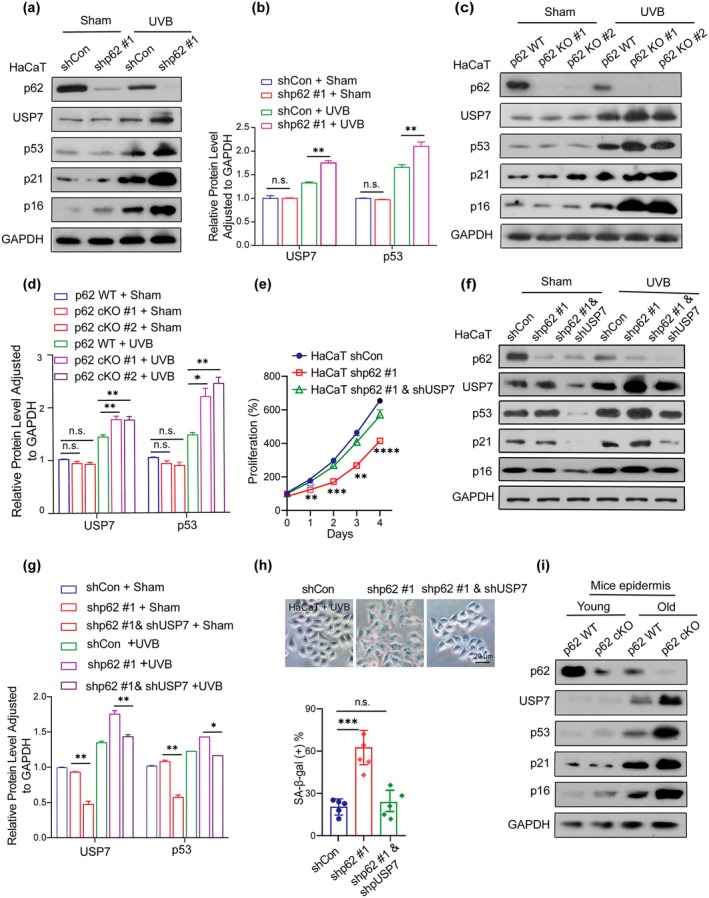
p62 inhibits the abundance of USP7 and USP7‐mediated p53/p21 pathway in response to inducing senescence. (a) Western blotting assay showed the expression of proteins in shCon and shp62 HaCaT cells; the cells were stimulated with 5 mJ/m^2^ UVB treatment (UVB) or without UVB treatment (Sham). (b) Quantification of USP7 and p53 protein expression in shCon and shp62 HaCaT cells. (c) Western blotting assay indicated the expression of proteins in both p62 wt and p62 knockout HaCaT cells with or without UVB treatment. (d) Quantification of USP7 and p53 protein expressions in HaCaT cells. (e) CCK8 assay showed the cell viability in shp62 HaCaT cells stably transfected with shRNA targeting p62 and USP7. (f) Western blotting assay showed expression of proteins in HaCaT cells with stable knockdown of p62 and USP7; the cells were stimulated with or without UVB treatment. (g) The protein expressions of USP7 and p53 were quantified and statistically evaluated. (h) Representative images of SA‐β‐gal staining were shown in p62 and USP7 stably knockdown HaCaT cells (top), and quantification of SA‐β‐gal‐positive cells was performed (bottom). Scale bar: 20 μm. (i) Western blotting assay measured expression of proteins in *p62* WT and *p62* cKO mice epidermis at 2 months old (young) and 20 months old (old). Data show mean ± SEM. Comparisons by two‐way ANOVA. ****p < 0.0001, ***p < 0.001, **p < 0.01, *p < 0.05, n.s., not significant.

Previous KEGG and GO analyses indicated that DNA replication and lysosome activity might play pivotal roles in the regulation of cellular senescence (Figure S1b,c). Moreover, USP7 and p53 were significantly upregulated in senescent human keratinocytes (Figure 1a). The ubiquitin–proteasome system (UPS) is essential for protein homeostasis and regulates aging through autophagy and ubiquitin signaling (Pozhidaeva and Bezsonova 2019) (Cui et al. 2020). In line with these findings, we found that USP7 expression increased in senescent cells and aging skin. Notably, both p62 knockdown and knockout upregulated USP7 and p53 protein levels in UVB‐treated keratinocytes (Figure 4a–d), suggesting that the autophagy–lysosome pathway regulates USP7 expression. In line with this, depletion of the autophagy component ATG7 also increased USP7 protein levels (Figure S4f). Furthermore, aging keratinocytes were found to inhibit autophagic degradation of USP7 (Figure S4g).

Previous studies suggested that USP7 plays a paradoxical role in regulating p53 functions. On the one hand, USP7 binds to and directly ubiquitinates p53, preventing its degradation. On the other hand, USP7 interacts with MDM2, stabilizing it by removing ubiquitin, thereby promoting MDM2‐mediated degradation of p53. Given the similar regulatory effects of USP7 and p53 on p62, we investigated whether p62 modulates p53 expression via USP7. To confirm whether p62 regulates senescence through the USP7‐mediated p53/p21 pathway, we stably knocked down USP7 in shp62 keratinocytes (Figure S4h). This abrogated the p62‐mediated inhibition of the p53/p21 pathway in response to UVB‐induced senescence and cell proliferation (Figure 4e–g). Consistently, USP7 knockdown in shp62 keratinocytes abolished the p62‐mediated inhibition of senescence (Figure 4h). Moreover, aged *p62* cKO mice showed pronounced upregulation of USP7 and p53 in the epidermis compared to *p62* WT mice (Figure 4i). These findings collectively suggest that p62 restrains the abundance of USP7 and the subsequent USP7‐mediated p53/p21 pathway in response to senescence induction.

### 2.5 p62 Interacts With USP7, PB1 Domain, and UBA Domain of p62 Are Required for USP7‐Ubl5 Binding

**FIGURE 5:**
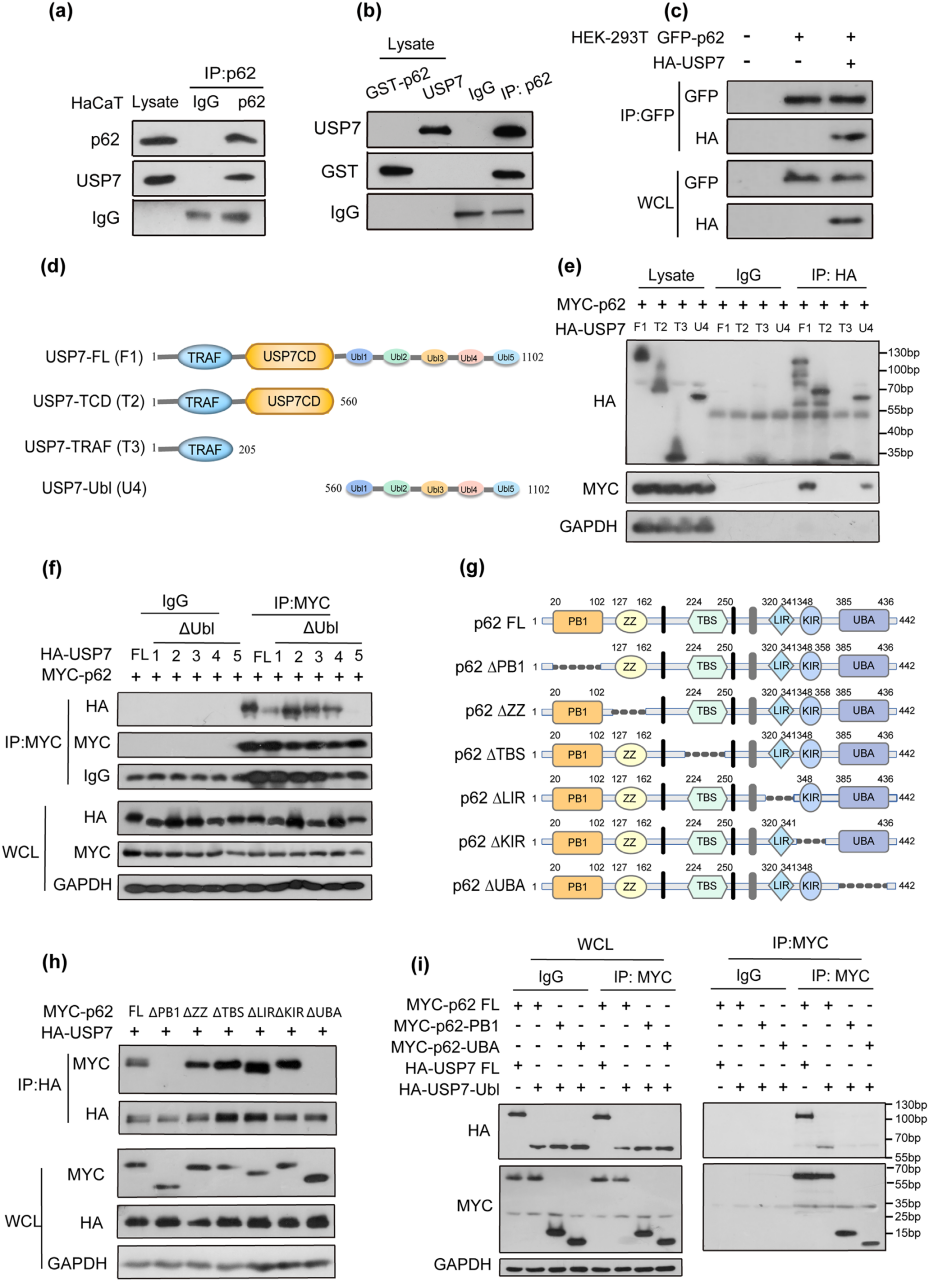
p62 interacts with USP7; the p62 PB1 domain and UBA domain are required for USP7‐Ubl5 binding. (a) Immunoprecipitation (IP) was carried out by incubating the lysate of HaCaT cells with p62 antibodies using rabbit IgG as the negative control. Immunoblotting (IB) was performed with antibodies against p62 and USP7. (b) Ectopically expressed GST‐USP7 was induced in the BL‐21 strain and subjected to GST pull‐down. GST‐p62 and USP7 proteins were cocultured in vitro for 48 h. GST‐p62 interacts specifically with USP7 through co‐IP assay with the anti‐p62 antibody. (c) HEK‐293 T cells were cotransfected with GFP‐p62 and HA‐USP7 for 24 h. The transfected cells were subjected to co‐IP assay with the anti‐GFP antibody. (d) Schematic representation of various deletion forms of HA‐USP7, including ubiquitin‐like (Ubl) domains 1 ~ 5 deletion mutant, HA‐USP7‐TCD; ubiquitin‐like (Ubl) domains 1 ~ 5 and crystal domain deletion mutants, HA‐USP7‐TRAF; N‐terminal TRAF‐like domain and crystal domain deletion mutants, HA‐USP7‐Ubl. (e) HEK‐293 T cells were cotransfected with full‐length MYC‐p62 and HA‐USP7 or USP7 deletion mutants for 24 h. The transfected cells were subjected to co‐IP assay with the anti‐HA antibody. F1, T2, T3, and U4 represent full‐length (FL), tumor necrosis factor receptor‐associated factors and catalytic domain (TCD), tumor necrosis factor receptor‐associated factors (TRAF) and C‐terminal tandem ubiquitin‐like (Ubl) of USP7. (f) HEK‐293 T cells were cotransfected with full‐length MYC‐p62 and full‐length or Ubl1–Ubl5 deletion mutants of HA‐USP7 for 24 h. The transfected cells were subjected to co‐IP assay with the anti‐MYC antibody. (g) Schematic representation of various deletion forms of GFP‐p62, including PB1, ZZ, TBS, LIR, KIR, and UBA domain deletion mutants, ΔPB1, ΔZZ, ΔTBS, ΔLIR, ΔKIR, and ΔUBA. (h) HEK‐293 T cells were cotransfected with the p62‐PB1 domain, p62‐UBA domain, and USP7‐Ubl domain for 24 h. The cells were subjected to co‐IP assay with the anti‐HA antibody. (i) HEK‐293 T cells were cotransfected with full‐length HA‐USP7 and MYC‐p62 or p62 deletion mutants for 24 h. The transfected cells were subjected to co‐IP assay with the anti‐MYC antibody.

Our findings suggested that p62 suppressed USP7 abundance in senescent keratinocytes, where autophagic degradation of USP7 was impaired (Wang et al. 2017). To investigate whether p62 interacts with USP7, we first examined their colocalization in keratinocytes. Immunofluorescence staining results revealed that p62 and USP7 colocalized in the cytoplasm (Figure S5a,b). Co‐immunoprecipitation (Co‐IP) confirmed the binding between endogenous p62 and USP7 in HaCaT cells (Figure 5a and Figure S5c), and in vitro assays showed a direct interaction (Figure 5b). Further analysis in HEK293 cells confirmed the binding between exogenous HA‐USP7 and GFP‐p62 (Figures 5c and S5d). Collectively, these findings provide compelling evidence that p62 physically interacts with USP7, suggesting a direct role for p62 in modulating USP7 abundance and function.

This domain diversity of p62 allows it to interact with ubiquitinated substrates via its UBA domain and with the proteasome through its PB1 domain (Islam et al. 2018). The above data suggested a direct interaction between p62 and USP7. The full‐length USP7 consists of three key domains: the tumor necrosis factor receptor‐associated factors (TRAF) domain (amino acids 62–205), the catalytic domain (amino acids 208–560), and the C‐terminal tandem ubiquitin‐like (Ubl) domain (amino acids 560–1102) (Wang et al. 2019) (Figure 5d). As reported, the TRAF domain binds MDM2 and p53, contributing to the nuclear localization of USP7. The catalytic domain mediates ubiquitin binding and deubiquitination of the substrate. Besides, the USP7 truncation (amino acids 208–1102) performed similar activity to the full‐length protein. p62, a multifunctional scaffold protein, plays an integral role in maintaining cellular homeostasis. These domains include the N‐terminal Phox1 and Bem1p (PB1) domain, a zinc finger (ZZ), a tumor necrosis factor receptor‐associated factor 6 (TRAF6)‐binding (TB) motif, an LC3‐interacting region (LIR), a Keap1‐interacting region (KIR), and a ubiquitin‐associated (UBA) domain (Manley et al. 2013). To identify the domain of USP7 that binds p62, we constructed various deletion forms of HA‐USP7, including USP7 FLS, USP7‐TCD, USP7‐TRAF, and USP7‐Ubl. Co‐IP assay revealed that p62 specifically binds the Ubl domain of USP7 (Figure 5e; Figure S5f), with p62 binding USP7 even in the absence of the Ubl5 domain (Figure 5f). To further elucidate the p62‐specific domain of the combination, we also constructed various p62 truncation mutants (Figure 5g). Given that p62 contains six distinct functional domains (PB1, ZZ, TB, LIR, KIR, and UBA) and is known to recognize ubiquitinated substrates via its UBA domain and interact with the proteasome through its PB1 domain (Islam et al. 2018), we investigated the role of these domains in USP7 binding. Notably, p62 mutants lacking either the PB1 or UBA domain failed to bind USP7 (Figures 5h and S5g). Furthermore, HEK‐293 T cells were cotransfected with the p62‐PB1 domain, p62‐UBA domain, and USP7‐Ubl domain. The results of the co‐IP assay with anti‐MYC antibody showed both the PB1 and UBA domains of p62 were ligand‐binding domains for the Ubl5 domain of USP7 (Figure 5i). Together, these results indicate that the PB1 and UBA domains of p62 are required for USP7–Ubl5 binding.

### 2.6 Mutations of Active Amino Acids Binding to p62 or USP7 Promote Cell Senescence

**FIGURE 6:**
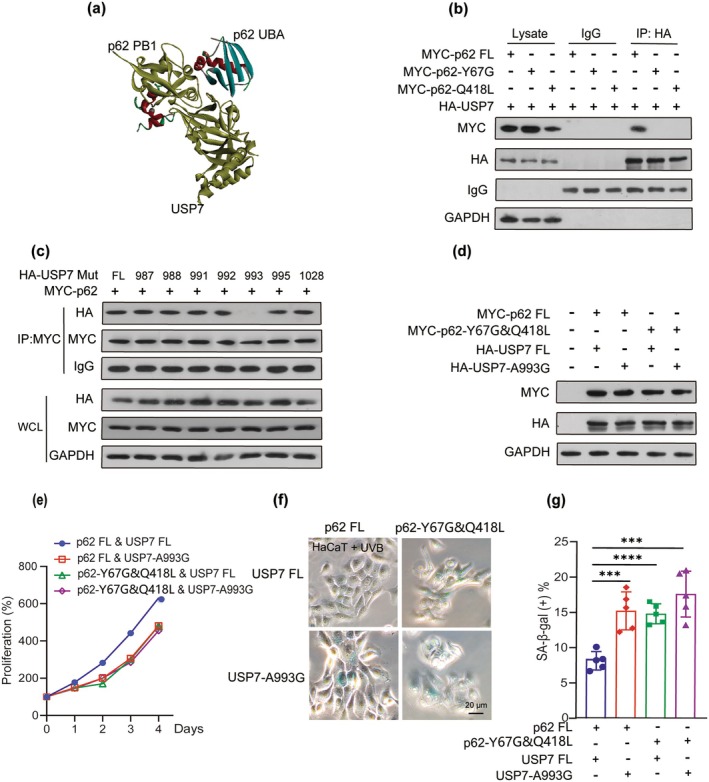
Mutations of active amino acids binding to p62 or USP7 promote cell senescence (a) Molecular docking analysis of the potential binding between p62‐PB1, p62‐UBA domain, and USP7‐Ubl domain. (b) HEK‐293 T cells were cotransfected with full‐length HA‐USP7 and MYC‐p62, MYC‐p62‐Y67G mutant, and MYC‐p62‐Q418L mutant for 24 h. The transfected cells were subjected to co‐IP assay with anti‐HA antibody. (c) HEK‐293 T cells were cotransfected with full‐length MYC‐p62 and full‐length or point mutants of HA‐USP7 for 24 h. The transfected cells were subjected to co‐IP assay with anti‐MYC antibody. (d) HaCaT cells were stably cotransfected with full‐length MYC‐p62 or MYC‐p62 mutant and HA‐USP7 or HA‐USP7 mutant. Immunoblot analysis of HA and MYC in cotransfected HaCaT cells. (e) CCK8 assay showed the cell viability in p62 or mutant and USP7 or mutant cotransfected HaCaT cells. (f) Representative SA‐β‐gal staining images were shown in UVB‐treated HaCaT cells. Scale bar: 20 μm. (g) Quantification of SA‐β‐gal‐positive cells was performed. Data show mean ± SEM. Comparisons by two‐way ANOVA. ****p < 0.0001, ***p < 0.001, n.s., not significant.

The molecular docking analysis identified potential amino acid interactions between the p62‐PB1 and p62‐UBA domains with the USP7‐Ubl domain (Figure 6a). Previous studies have shown that mutations K7A and D69A in the PB1 domain impaired the formation of MOAP‐1 and p62 aggregates under DEN conditions (Nakamura et al. 2010). Additionally, based on the reported DSK2 UBA‐ubiquitin complex, residue A341 of the p62 UBA‐ubiquitin complex and phosphorylation of S403 introduced a double negative charge into the p62 UBA‐ubiquitin interface (Berkamp et al. 2021; Jakobi et al. 2020). In the present study, mutations were introduced at the potential amino acids Y67 and D69 of the PB1 domain and seven sites in the UBA domain (Figure S5h,i). The Y67G mutation in the PB1 domain and Q418L mutation in the UBA domain abolished the binding activity of p62 (Figure 6b). Mutations at seven potential amino acids in the USP7 Ubl5 domain were also tested. Co‐IP assays showed that the A993Y mutation was critical for the binding activity of USP7 (Figure 6c). These results indicate that the active amino acids of the interaction between p62 and USP7 are Y67 of the PB1 domain or Q418 of the UBA domain and A993 of the Ubl5 domain.

To further investigate the contribution of active amino acids in the binding between p62 and USP7 during cellular senescence, HaCaT cells were cotransfected with wild‐type or mutant p62 and wild‐type or mutant USP7 (Figure 6d). Mutations at key sites, including Y67G and Q418L in p62 and A993Y in USP7, reduced keratinocyte viability (Figure 6e) and accelerated senescence of UVB‐treated keratinocytes (Figure 6f,g). Collectively, these data suggest that the coexpression of p62 and USP7 appears to exert an inhibitory effect on cell senescence, with the Y67 and Q418 amino acids in p62 and the A993 residue in USP7 playing pivotal roles in this regulatory process.

## 3 Discussions

The epidermis, as the body's outermost organ, plays a crucial barrier role. It is a rapidly proliferating and self‐renewing tissue, and its aging is often associated with structural and functional impairments (Strnadova et al. 2019). Despite recent advances in understanding dermal cell regulation in skin aging, the molecular mechanisms of epidermis aging remain incompletely understood. In this study, we found p62 expression is reduced in aging human or mouse skin and senescent cells, highlighting its essential role in maintaining skin structure and function. Keratinocyte p62‐deficient mice exhibited aging‐like symptoms such as decreased skin elasticity, wrinkles, increased water loss, and thinner epidermis. p62 reduction or depletion in keratinocytes significantly decreased promoted senscence and inhibited proliferation while p62 overexpression suppressed senescence and promoted proliferation. Thus, p62 acts as a negative regulator of both skin aging and cellular senescence.

Recent investigations have closely linked the deubiquitylase USP7 to lifespan regulation and cellular responses to environmental stressors such as starvation, oxidative stress, and heat stress (Cui et al. 2020). Inhibition of USP7 can selectively eliminate senescent cells by restoring p53 activity and suppressing SASP (He et al. 2020). The upregulation of USP7 and p300 mediates activation of the p53‐p21 pathway, leading to acceleratedpremature cellular senescence in chronic obstructive pulmonary disease patients (Zeng et al. 2022). Our results showed abundant expression of USP7 in both aging mice skin and senescent keratinocytes. It has been reported that USP7 can regulate aging depending on the autophagy and ubiquitin signaling pathways (Cui et al. 2020). we further found that aging keratinocytes exhibited impaired autophagic degradation of USP7, suggesting a potential mechanism through which USP7 accumulation contributes to epidermal aging.

p62 is a scaffold protein that promotes the formation and degradation of ubiquitinated aggregates via its PB1 and UBA domains (Vargas et al. 2023). Upon senescence induction, the p62‐GATA4 interaction is decreased, stabilizing GATA4 and promoting senescence (Xiong et al. 2022). UVB‐induced DNA and protein damage, a key driver of aging, exacerbates these effects (Salminen et al. 2022). Our findings suggest that complex formation between p62 and USP7 is reduced in senescent cells. We found that the p62–USP7 interaction is diminished in senescent cells, promoting USP7 degradation via autophagy.

p62 comprises multiple functional domains, including PB1, ZZ, TB, LIR, KIR, and UBA (Manley et al. 2013). The PB1 domain of p62 interacts with other protein kinases, facilitating its oligomerization with other PB1‐containing proteins (Islam et al. 2018). Meanwhile, the UBA domain of p62 binds to ubiquitinated cargo destined for lysosomal degradation via autophagy (Islam et al. 2018). Many proteins have been identified as potential substrates and binding partners of USP7, most of which play important roles in diverse biological processes. The PB1 and UBA domains of p62 are required for binding USP7 via its Ubl5 domain. However, previous studies have considered the PB1 and UBA domains of p62 as relatively independent functional regions, suggesting a potentially novel binding mechanism between p62 and other proteins. The underlying reason for this dual‐domain requirement in USP7 binding remains unclear. It is unclear why the PB1 domain and UBA domain of p62 were required for the interaction with USP7. Structurally, p62 exhibits both acidic and basic surfaces (AB‐type) and contains a PB1 domain capable of homopolymerizing into flexible quaternary structures, thereby expanding its functional versatility and adaptability (Ciuffa et al. 2015). This property allows p62 to act as a molecular hub, orchestrating interactions with multiple proteins. Additionally, self‐interactions within the UBA domain promote the formation of stable p62 filaments, which may be disrupted by polyubiquitin (Feng et al. 2022). Conversely, ubiquitinated cargo enhances the stabilization of these filaments, promoting the formation of larger p62 condensates (Zaffagnini et al. 2018). The USP7 full‐length protein comprises an N‐terminal polyglutamine stretch, the TRAF domain, a catalytic domain, and a C‐terminal tandem Ubl domain containing Ubl1 to Ubl5 (Wang et al. 2019). Notably, the complex formed by Ubl4 and Ubl5 is sufficient to activate USP7 both in vitro and in vivo (Peng et al. 2023). Based on these findings, we hypothesize that the filamentous structures formed by the PB1 domain of p62 may facilitate its interaction with the Ubl5 domain of USP7. Subsequently, self‐associations within the UBA domain of the p62 filaments may further stabilize this interaction. However, further investigation is required to validate this hypothesis and elucidate the precise molecular mechanism underlying this interaction.

Owing to the interaction between p62 and USP7, we further investigated the functional significance of key residues within the PB1 or UBA domain of p62 and the Ubl5 domain of USP7 in the regulation of cellular senescence. Previous studies have shown that the K7A and D69A mutants failed to form MOAP‐1 and p62 aggregates under DEN conditions (Nakamura et al. 2010). Besides, A341 of the p62 UBA‐ubiquitin complex is based on the reported DSK2 UBA‐ubiquitin complex. Phosphorylation of S403 introduces a double negative charge into the p62 UBA–ubiquitin interface (Berkamp et al. 2021; Jakobi et al. 2020). The molecular docking analyses further revealed potential amino acid interactions between the PB1 and UBA domains of p62 and the Ubl5 domain of USP7. Functional assays confirmed that the critical residues mediating p62–USP7 binding are Y67 of the PB1 domain or Q418 of the UBA domain and A993 of the Ubl5 domain. Further investigation explored the role of the active sites between p62 and USP7 binding in cellular senescence and whether Y67G and Q418L mutants of p62 or A993G mutant of USP7 promoted cell senescence and decreased cell viability of keratinocytes. Notably, these mutations promoted cellular senescence and reduced cell viability, highlighting the inhibitory role of p62–USP7 interaction in senescence regulation. Collectively, the combination of p62 and USP7 inhibits cell senescence, in which Y67 and Q418 amino acids of p62 and A993 of USP7 play key roles.

In conclusion, our results revealed that p62 plays a significant role in skin aging. Deletion of p62 can accelerate skin aging phenotypes and promote keratinocyte senescence. p62 inhibited the USP7‐mediated p53/p21 pathway in response to UVB‐induced cellular senescence of the epidermis. p62 interacts with USP7 to downregulate the abundance of USP7 in response to cellular senescence. The PB1 domain and UBA domain of p62 are both required for the Ubl5 domain of USP7 binding. Y67 and Q418 amino acids of p62 and A993 of USP7 play key roles in inhibiting cellular senescence. This suggests that p62 might be a potential therapeutic target to improve the function of keratinocytes and contribute to delayed skin aging.

## 4 Experimental Procedures

### 4.1 Mice Feeding and Treatments

*Sqstm1* WT mice (*Sqstm1* flox/flox) and K14‐Cre mice were the kind gifts of Dr. Toru Yanagawa, University of Tsukuba, Japan. All the animals were housed in an environment with a constant temperature and humidity. Autoclaved food and water were provided ad libitum. All animals were fed until 2, 6, 12, 16, and 20 months old, with experiments conducted following approved protocols by the Animal Welfare and Ethics Committee of China Pharmaceutical University (2021‐09‐040).

### 4.2 Human Samples

Skin specimens were obtained from healthy individuals of different ages at the Chinese Academy of Medical Sciences and Peking Union Medical College. This study was approved by the Ethnic Committee of the Chinese Academy of Medical Sciences and Peking Union Medical College (2020KY‐012). Informed consent was obtained from all participants before sample collection.

### 4.3 Cell Culture

HEK‐293 T cells and IMR‐90 cells were purchased from the American Type Culture Collection. HEK‐293 T cells, HaCaT cells, and primary keratinocytes were cultivated in DMEM medium. All the media were supplemented with 10% fetal bovine serum and 1% penicillin/streptomycin. IMR‐90 cells were cultured in MEM medium with 10% fetal bovine serum and 1% penicillin/streptomycin. Cells were cultured at 37°C in a humidified incubator containing 5% CO_2_.

H‐Ras^V12^, IR, and UVB‐induced cellular senescence models were constructed, and the cellular senescence model was obtained after H‐Ras^V12^ lentiviral infection of the target cells for 48 h; the cellular senescence model was obtained by 10G IR irradiation of the target cells followed by 48 h of incubation; and the HaCaT cells were irradiated by 5 mJ/cm^2^ UVB for 5 days with an interval of 24 h followed by 3 days of incubation. A cell senescence model was obtained.

The cultivation of murine primary keratinocytes and fibroblasts: Newborn nude mice were immersed in 75% ethanol twice, 2 min each time. The limbs and tails were removed, and the skin was carefully excised along the dorsal midline, extending toward the nose. The skin was then inverted over the forelimbs and washed thoroughly with HBSS (140 mM NaCl, 5 mM KCl, 0.44 mM KH_2_PO_4_, 0.40 mM Na_2_HPO_4_, 5.56 mM glucose, and 4.17 mM NaHCO_3_). The excised dermis was placed dermis‐side down in 2.5% trypsin/HBSS and incubated at 37°C for 1 h to separate the epidermis from the dermis. The epidermis was dissected into small fragments and subjected to further digestion with trypsin for 30 min at 37°C to obtain a single‐cell suspension. The suspension was filtered through a 40‐μm cell strainer and collected in new 15 mL tubes. At the same time, the intact dermis was immersed in normal trypsin for 2 h at 37°C to digest into single cells and filtered through a 40‐μm cell strainer into a new 15 mL tube. Centrifuge the cells at 500 r for 5 min. Aspirate the supernatant, resuspend the pellet with DMEM medium in a 6 cm cell culture dish, and perform culture as previously described. Primary fibroblasts and keratinocytes were used to verify the mouse genotype. Murine primary fibroblasts and keratinocytes were used to verify the mouse genotype. Murine primary keratinocytes were subjected to oncogene (H‐Ras^V12^)‐, IR‐induced senescence, or replicative senescence to assess the expression of p62.

### 4.4 Plasmid Construction and Transfection

Constructs coding for p62 (or USP7) and their point mutants were cloned in the pCMV vector for transient expression and the pLenti v2 vector for permanent expression. The p62 and USP7 cDNA sequences were inserted into the pGEX‐6P‐1 vector with a GST tag. The shRNA and gRNA sequences targeting genes such as p62 and p53 were inserted into the pLKO.1 or LentiCRISPR vector for knockdown and knockout targeting genes. HEK‐293 T, IMR‐90, and HaCaT cell transfection were performed using polyplus transfection according to procedures recommended by the manufacturer. The sequences of the primers used for gene amplification are listed in Table S2.

### 4.5 Site‐Directed Mutagenesis

Mutations of plasmids were performed using a Mut Express II kit according to the manufacturer's guidelines. The primers used are listed in Table S3.

### 4.6 Real‐Time Polymerase Chain Reaction (RT‐qPCR) Analysis

Total RNA was isolated from tissues and cells using RNAiso Plus (cDNA was synthesized with 1 μg of total RNA using HiScript II Q RT SuperMix). Real‐time PCR was completed on a LightCycler 480 Instrument II (Roche, Basel, Switzerland). The threshold cycle number for each sample was determined in triplicate and normalized to GAPDH. All primers are listed in Table S1.

### 4.7 Western Blotting Assay

Cell and tissue samples were prepared using RIPA buffer containing inhibitors for proteases and phosphatases. Samples were analyzed by SDS‐PAGE and transferred to membranes with appropriate antibodies.

### 4.8 Proteomics Sample Preparation

A procedure for cellular lysis, protein digestion, and peptide purification was conducted. Cells were lysed with trifluoroacetic acid (TFA) and then neutralized with Tris–HCl. Tris (2‐carboxyethyl) phosphine (TCEP) and chloroacetamide (CAA) were added, mixed, and incubated. Proteins were digested with trypsin at 37°C for 16 h before termination with TFA, and peptides were desalted using a C18 tip.

### 4.9 Coimmunoprecipitation (Co‐IP) Assays

After transfection or treatment, the cells were lysed in IP buffer (1% NP‐40 pH = 7.4, 50 mM Tris–HCl, 150 Mm NaCl, 1 mM EDTA, and 1 mM PMSF) on ice for 1 h. The lysate was cleared by centrifugation at 14000 g at 4°C for 20 min. Cell lysates underwent immunoprecipitation with antibodies overnight at 4°C. Normal IgG served as a negative control. Protein A/G agarose beads were added and incubated for 2 more hours. The beads were washed thrice with IP buffer before suspension in 40 μL IP buffer plus 8 μL SDS‐PAGE loading buffer. The proteins were boiled at 120°C for 10 min and analyzed by SDS‐PAGE followed by immunoblotting using appropriate antibodies.

### 4.10 Immunofluorescence

The cells or slices were immersed in 4% paraformaldehyde for 20 mins, washed thrice in PBS, and then permeabilized with 0.5% Triton X‐100/PBS for 30 mins. They were blocked in PBST/5% BSA for 2 h and incubated overnight at 4°C with primary antibodies. After rinsing three times with PBST, they were counterstained with secondary antibodies/BSA‐PBST for 1 h in the dark, washed with PBST, stained with DAPI for 10 min, and mounted on slides using a mounting medium. The primary antibodies are listed in the key resources table.

### 4.11 Protein Expression and Purification

All recombinant proteins were expressed in *E. coli* BL21. Protein expression was induced at OD_600_ = 0.8 with 0.1 mM IPTG for 4 h at 37°C. Then cells were harvested and washed with STE buffer (100 mM NaCl, 10 mM Tris–HCL, 1 mM EDTA, and pH = 8.0), lysed in lysis buffer (200 μL 50 mg/mL lysozyme, 200 μL 10%NP‐40, 0.1 mM MgCl_2_, 40 μL 10 μg/μl D‐Nase, and 1 mM PMSF), and supplemented sufficiently. Ultrasonic disruption of cells was performed. Lysates were cleared by centrifugation at 14000 g at 4°C for 15 min, and the supernatant was added to 50 mL tubes containing 500 μL Pierce Glutathione Agarose. The GST beads were washed three times with 10 mL STE buffer each time. Then, proteins and GST beads were rotated overnight, and bound proteins were eluted with elute buffer (30 mM L glutathione reduced, 200 mM Tris–HCl). Protein supernatant was collected by centrifugation at 2000 rpm at 4°C for 2 min and stored at −80°C.

### 4.12 SA‐β‐Gal Staining

The slides were washed with PBS, fixed with 4% paraformaldehyde at room temperature for 5 min, added SA‐β‐gal staining solution, and incubated overnight at 37°C without CO_2_. After incubation, the samples were mounted using a mounting solution and observed with an ordinary light microscope.

### 4.13 CCK8

Cells in good growth condition were harvested and resuspended at a concentration of 1×10^5^ cells/mL; 100μL per well was added into a 96‐well cell culture plate. Cultivate the cells according to the experimental requirements. 10 ul CCK‐8 solution was added to a 96‐well cell culture plate and incubated for 0.5–4 h in a 37°C incubator. The absorbance of cells was determined.

### 4.14 Flow Cytometry

The primary keratinocytes were seeded into six‐well plates and underwent appropriate treatment. Subsequently, the residual medium was rinsed away with chilled PBS buffer. EDTA digestion was performed on ice. The cells were then collected via centrifugation at 300 g for 5 min at 4°C. The supernatant was discarded, and 100 μL of I/C fixation solution was added to the pellet. This mixture was incubated for 40 min at room temperature. After washing once or twice with permeabilization buffer, 200 μL of transmembrane solution was added to the cells and incubated for another 30 min at room temperature. The cells were then incubated with an antibody for 1 h on ice, and protected from light. After washing twice with chilled PBS, the cells were resuspended in 300 μL of PBS. Finally, the cells were analyzed using flow cytometry (NovoCyte, Agilent).

### 4.15 Measurement of Moisture and Transepidermal Water Loss (TEWL)

Moisture and TEWL were measured on the ears of mice with the CK‐MPA4 Corneometer CM825 and Tewameter TM300.

### 4.16 ELISA

The protein levels of SASP factors from mouse serum or skin were determined with ELISA kits following the manufacturers' protocols.

### 4.17 Hematoxylin and Eosin (H&E) and Immunohistochemistry Staining

The tissues underwent fixation in 4% PFA, paraffin embedding, sectioning at 5 μm thickness, deparaffinization, and staining with hematoxylin and eosin. Prior to immunostaining, skin samples were incubated with 1% H_2_O_2_ to block residual peroxidase activity. They were subsequently treated with secondary IgG‐HRP‐conjugated antibody (1:200) for 1 h at room temperature, followed by staining using ABC and DAB reagents. Finally, sections were mounted with neutral balsam.

### 4.18 Masson Staining

Tissues were deparaffinized, immersed in Bouin's fixative solution (1% picric acid, 9% formaldehyde, and 5% glacial acetic acid) in a 56°C water bath for 15 min, and rinsed lightly with tap water until yellow disappears. The tissues were stained for 5 min with hematoxylin, then lightly rinsed for 5 min. Lastly, the tissues were stained with Dietrich scarlet acid, complex (phosphotungstic and phosphomolybdic), and aniline blue, immersed in 1% glacial acetic acid for 2 min, dehydrated, and covered with neutral balsam.

### 4.19 Molecular Simulation Analysis

Proteins form disulfide bonds through selective amino acid mutations that may enhance protein stability. The Predict Disulfide Bridges tool attempts to establish disulfide bonds between pairs of amino acids, maintaining the best geometric spatial distance between them, while the following factors may affect the stability of the established disulfide bonds, such as steric hindrance, solvent–soluble surface area, depth of amino acid burial, volume alteration, sequence spacing, and change in temperature factor. Amino acid sites with high disulfide bond affinity were obtained.

### 4.20 Bioinformatics Analysis

We entered gene expression profiling datasets for senescence in the GEO database and gene lists in senescence‐associated pathways collected from GO and KEGG terms. All statistical analyses were performed in the R programming environment (version 4.0.2).

### 4.21 Statistic

Statistical analyses were performed with GraphPad Prism software. two‐tailed Student's *t*‐test were used to compare 2 groups. Comparisons among multiple groups were performed with ANOVA. *p* < 0.05 was considered significant.

## Author Contributions

L.C. and X.W. designed and carried out the experiments and wrote the manuscript. Y.W. and Q.Y. analyzed data and performed statistical analysis. Y.L. and Y.Z. helped proofread the manuscript. H.H. provided quantitative proteomics analysis. H.Y. and Y.Y. prepared the material for experiments. Y.H. conceived the idea and reviewed the manuscript. L.Q. administered and supervised the project. All the authors read and approved the final manuscript.

## Conflicts of Interest

The authors declare no conflicts of interest.

## Supporting information


Figures S1‐S5.

Tables S1‐S3.

## Data Availability

The data that supports the findings of this study are available in the supplementary material of this article.
