# Supplementary material for: SQSTM1/p62 Orchestrates Skin Aging via USP7 Degradation
Source: Aging Cell. 2025 May 8;24(7):e70078. doi: 10.1111/acel.70078 (PMC12266749; doi:10.1111/acel.70078)
Supplement: Supplementary file 1 — Figures S1‐S5. [file ACEL-24-e70078-s002.docx]

**Supplemental Figures**

**SQSTM1/p62 orchestrates skin aging via USP7 degradation**

Liu Chen, Xiaoping Wang, Yuchen Wang, Qingxin Yao, Yunyao Liu, Yongcheng Zhu, He Huang, Hedan Yang, Yin Yang, Yuan He, and Lei Qiang

**FIGURE S1**


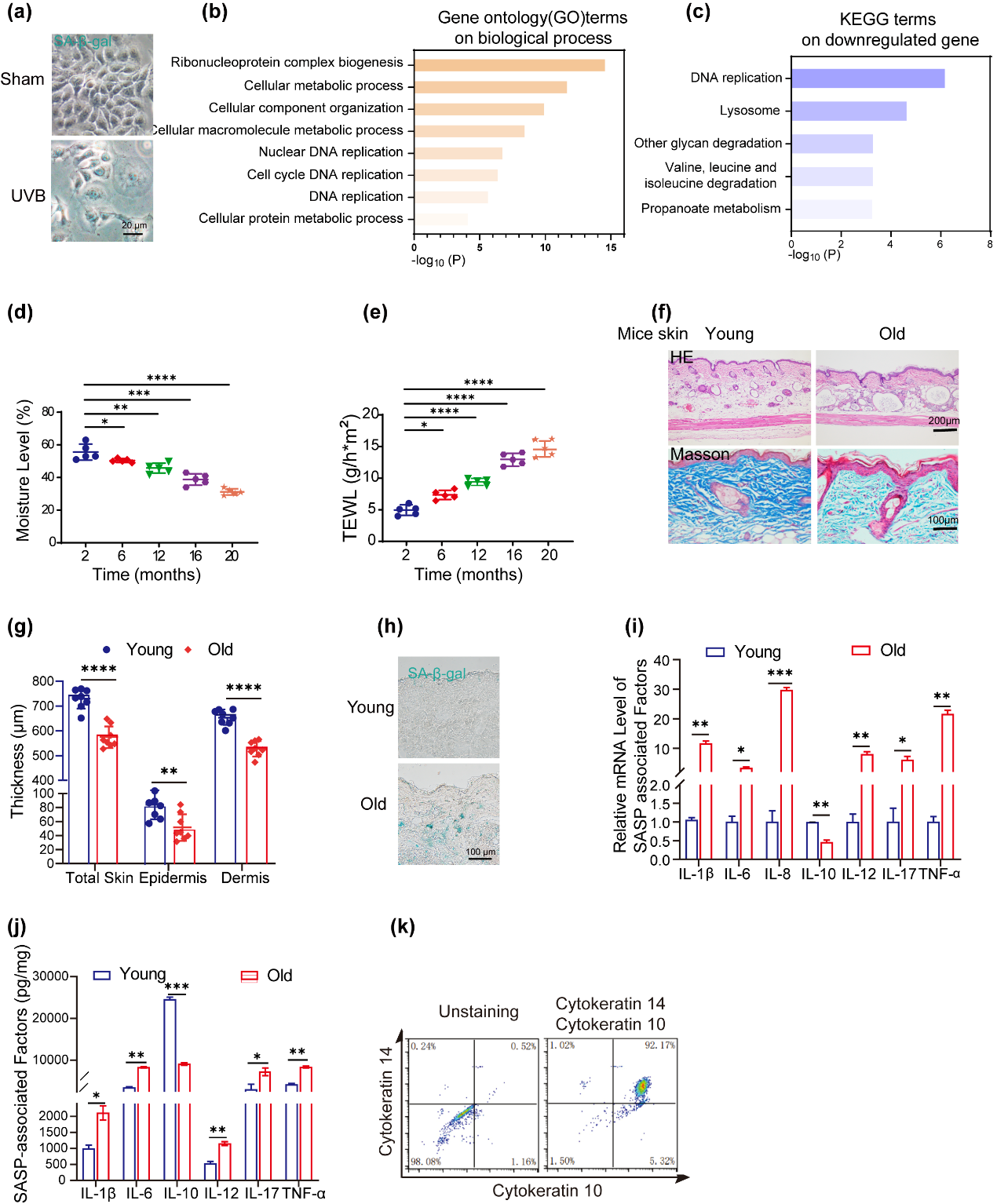


**FIGURE S1**

p62 is reduced in senescent cells and aging skin. (a) Representative SA-β-gal staining images were shown in sham and UVB-induced senescent human keratinocytes. Scale bar: 20 μm. (b) Gene GO terms enriched pathway in nonsenescent and senescent human keratinocyte cells (P< 0.05, n=3). (c) Gene KEGG enriched pathway in nonsenescent and senescent human keratinocyte cells (P< 0.05, n=3). (d) Moisture and (e) Transepidermal water loss (TEWL) measurements were performed to detect moisture content and percutaneous water loss of the stratum corneum to retain water of 2, 6, 12, 16, and 20-month-old mice (n=5). (f) Representative HE and Masson trichrome-stained images of skin Scale bar: 200 μm and 100 μm. (g) Quantitative analysis of skin epidermal and dermal thickness. (h) Representative images of SA-β-gal staining showed SA-β-gal expression detected in 2-month-old (Young), and 20-month-old (Old) mice skin. Scale bar: 100 μm. (i) The expression of senescence-associated secretory phenotype (SASP) factors was quantified in the epidermis of young and old mice. (j) ELISA assay measured secretion of SASP factors in the epidermis of young and old mice skin. (k) Flow cytometric analysis of murine primary keratinocytes. Data show mean ± SEM. Comparisons by unpaired t test. ****p<0.0001, ***p<0.001, **p<0.01, *p<0.05.

**FIGURE S2**


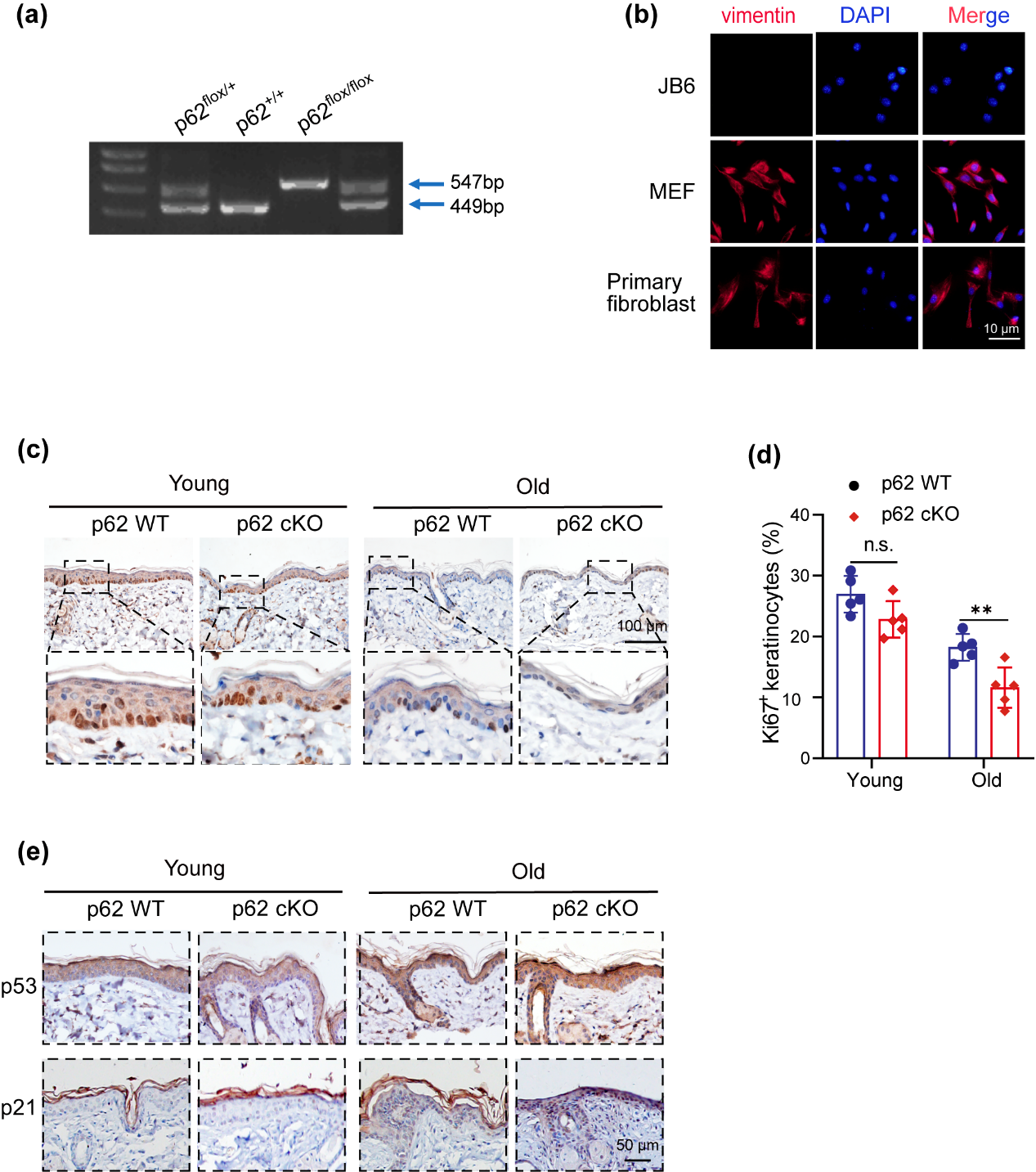


**FIGURE S2**

Epidermal p62 deficiency accelerates mice skin aging. (a) Genotyping identification of epidermal p62 deficiency SKH1 mice. (b) JB6, MEF and murine primary fibroblasts were stained with vimentin (green) and DAPI (blue). Scale bar: 10 μm. (c) Representative images of Ki67 staining were detected in p62 WT and p62 cKO mice skin at 2 months old (Young) and 20 months old (Old). Scale bar: 100 μm. (d) Quantitative analysis of Ki67^+^ keratinocytes was performed. (e) Representative p53 and p21 staining images were detected in p62 WT and p62 cKO mice skin at 2 months old (Young) and 20 months old (Old). Scale bar: 50 μm. Data show mean ± SEM. Comparisons by unpaired t test. **p<0.01, n.s., not significant.

**FIGURE S3**


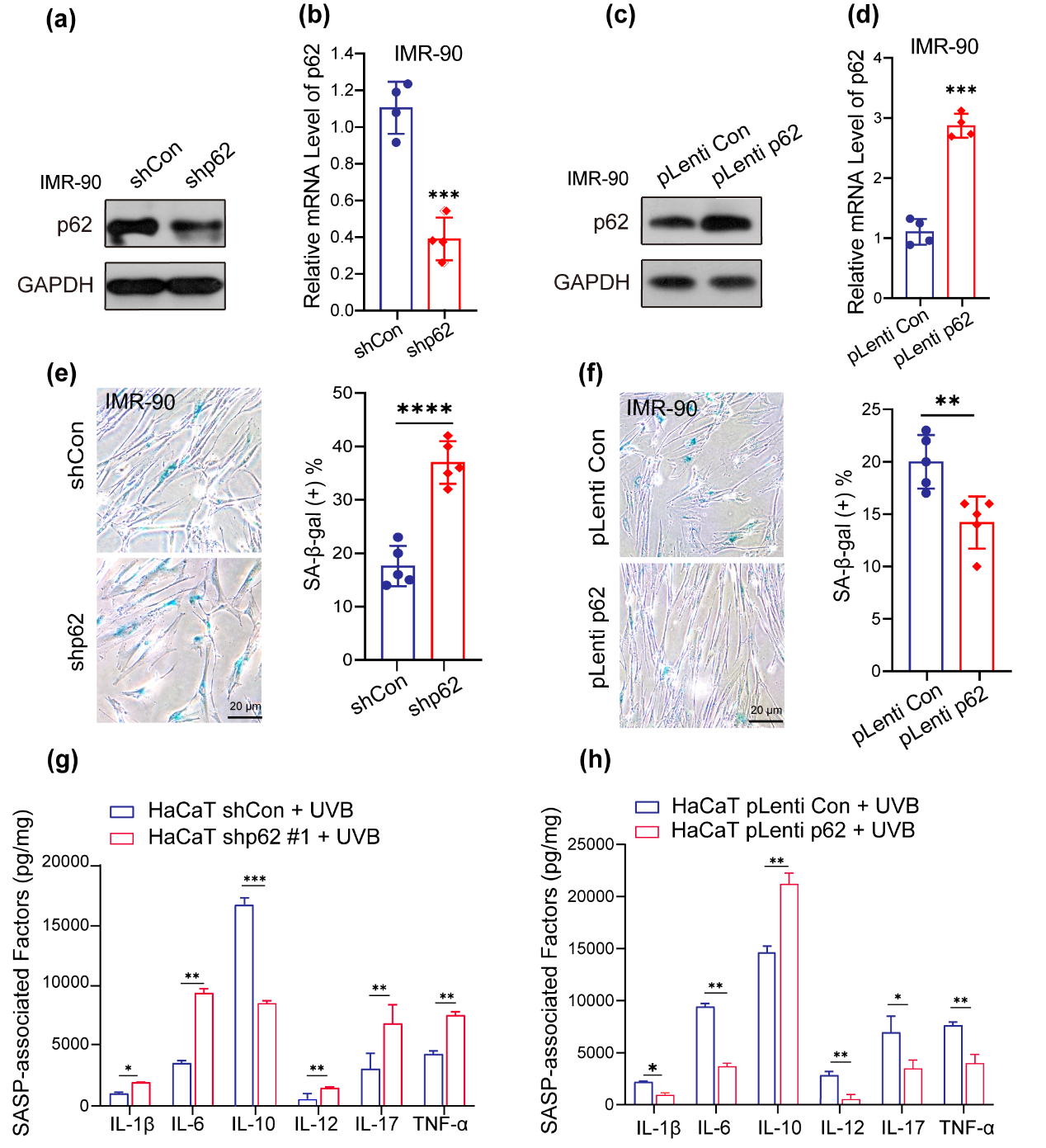


**FIGURE S3**

p62 regulates cellular senescence. (a) Immunoblot analysis of p62 and GAPDH in IMR-90 cells stably transfected with vector and shRNA targeting p62. (b) Expression of p62 in shCon and shp62 IMR-90 cells was measured by RT-qPCR. (c) Immunoblot analysis of p62 and GAPDH in pLenti Con and pLenti p62 IMR-90 cells. (d) Expression of p62 in pLenti Con and pLenti p62 IMR-90 cells was measured by RT-qPCR. (e) Representative SA-β-gal staining images were shown in shCon and shp62 IMR-90 cells (left), and quantitative analysis of SA-β-gal positive cells was performed (right). Scale bar: 20 μm. (f) Representative SA-β-gal staining images were shown in pLenti Con and pLenti p62 IMR-90 cells. Scale bar: 20 μm. (g) Secretion of SASP factors was measured by ELISA assay in p62 stably knock-down HaCaT cells. (h) Secretion of SASP factors was measured by ELISA assay in p62 overexpression HaCaT cells. Data show mean ± SEM. Comparisons by Two-way ANOVA for g and h. Comparisons by unpaired t test for b and d-f. ****p<0.0001, ***p<0.001, **p<0.01.

**FIGURE S4**


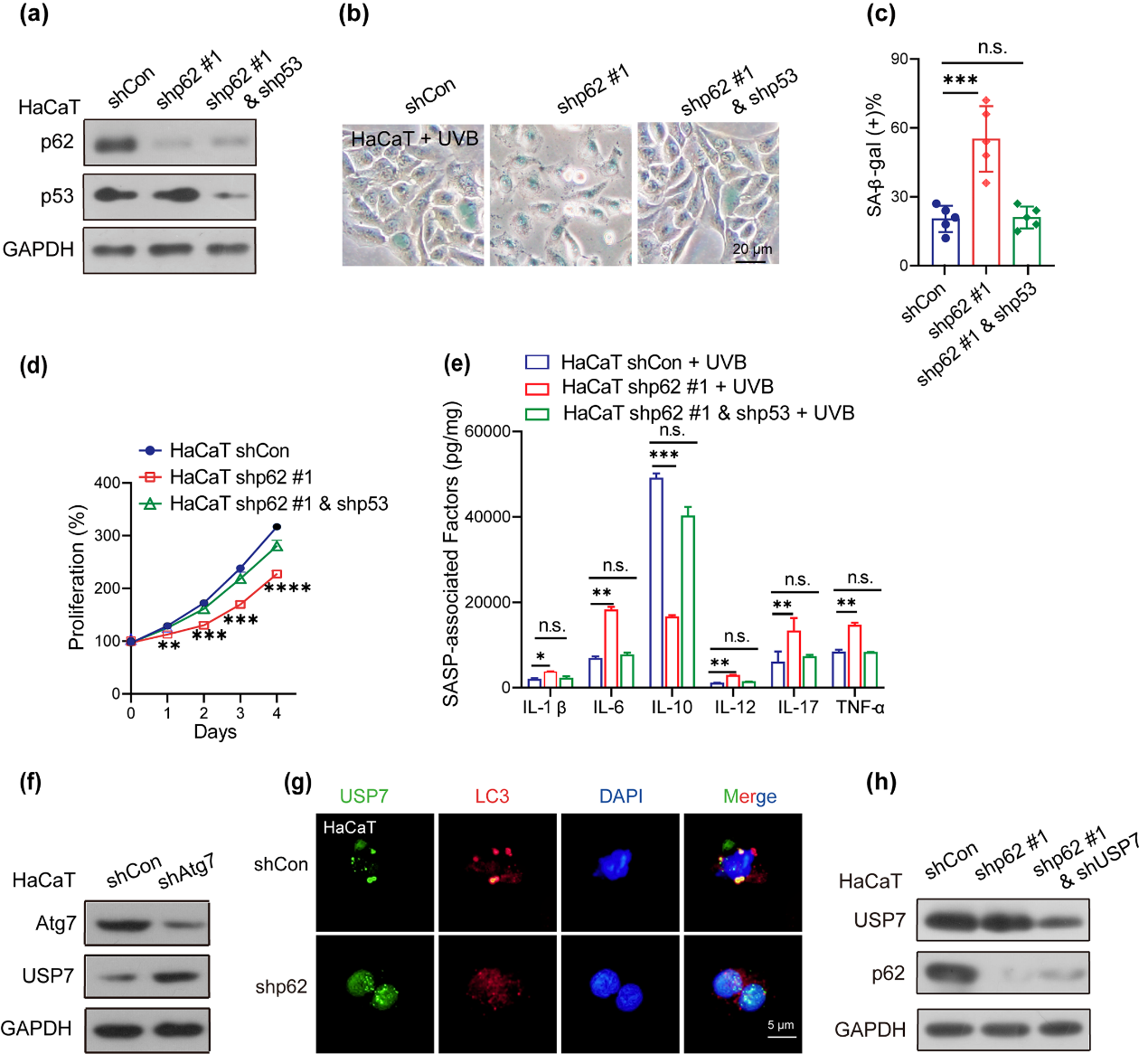


**FIGURE S4**

p62 inhibits the abundance of USP7 and USP7-medicated p53/p21 pathway in response to inducing-senescence. (a) Expression of p53 in p62 knockdown HaCaT cells stably transfected with shRNA targeting p53 was detected by immunoblotting analysis. (b) Representative images of SA-β-gal staining were shown in UVB-treated HaCaT cells. Scale bar: 20 μm. (c) Quantitative analysis of SA-β-gal positive cells was performed. (d) CCK8 assay showed the cell viability in shp62 HaCaT cells stably transfected with shRNA targeting p53. (e) ELISA analysis of SASP factor levels in p62 and p53 stably knock-down HaCaT cells. (f) HaCaT cells were stably transfected with shRNA targeting Atg7, and proteins were analyzed by Western blotting. (g) Representative images of HaCaT cells stained with USP7 (green), LC3 (red), and DAPI (blue). Scale bar: 5 μm. (h) An immunoblotting assay detected the expression of USP7 in shp62 HaCaT cells stably transfected with shRNA targeting USP7. Data show mean ± SEM. Comparisons by Two-way ANOVA. ****p<0.0001, ***p<0.001, **p<0.01, ns, not significant.

**FIGURE S5**


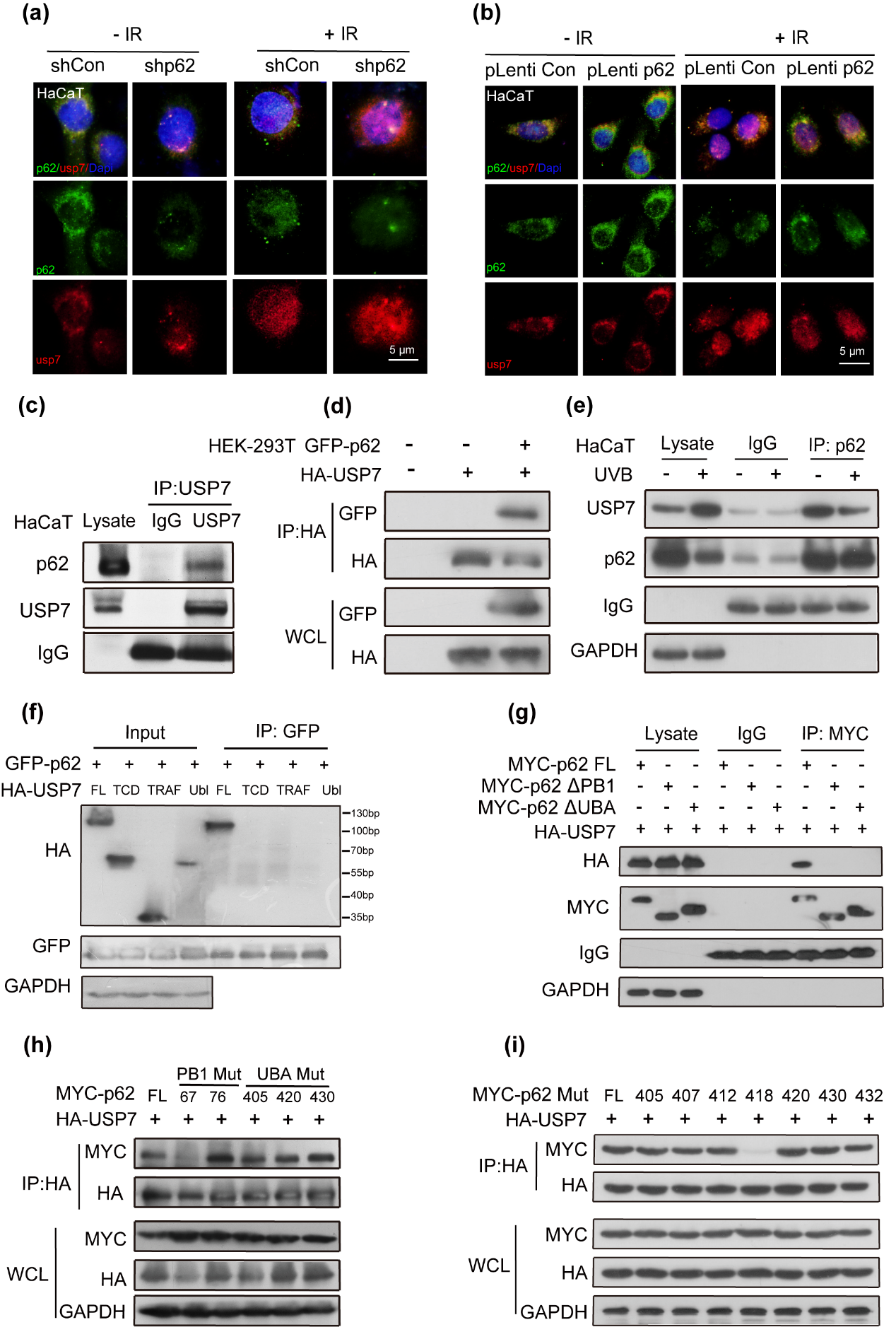


**FIGURE S5**

p62 interacts with USP7, p62 PB1 domain and UBA domain are required for USP7-Ubl5 binding. (a, b) Representative photomicrographs of HaCaT cells stained with p62 (green), USP7 (red) and DAPI (blue). The cells were cultured for 5 days after non-treated (-IR) or 10 Gy IR-treated (+IR). Scale bar: 5 μm. (c) Immunoprecipitation (IP) was carried out by incubating the lysate of HaCaT cells with USP7 antibodies using rabbit IgG as the negative control. Immunoblotting (IB) was performed with antibodies against p62 and USP7. (d) HEK-293T cells were co-transfected with GFP-p62 and HA-USP7 for 24 h. The transfected cells were subjected to co-IP assay with anti-HA antibody. (e) HaCaT cells were stimulated with non-treated (-UVB) or 5 mJ/m^2^ UVB-treated (+UVB) for 5 times. The cultured cells were subjected to co‐IP assay with anti-p62 antibody. (f) HEK-293T cells were co-transfected with full-length GFP-p62 and HA-USP7 or USP7 deletion mutants for 24 h. The transfected cells were subjected to co‐IP assay with anti-GFP antibody. (g) HEK-293T cells were co-transfected with full-length HA-USP7 and MYC-p62 FL, MYC-p62 ΔPB1 and MYC-p62 ΔUBA for 24 h. The transfected cells were subjected to co‐IP assay with anti-MYC antibody. (h) HEK-293T cells were co-transfected with full-length HA-USP7 and MYC-p62 mutants of p62 for 24 h. The transfected cells were subjected to co‐IP assay with anti-HA antibody. (i) HEK-293T cells were co-transfected with full-length HA-USP7 and MYC-p62 mutants of p62 for 24 h. The transfected cells were subjected to co‐IP assay with anti-HA antibody.
