# Supplementary material for: SQSTM1/p62 Orchestrates Skin Aging via USP7 Degradation
Source: Aging Cell. 2025 May 8;24(7):e70078. doi: 10.1111/acel.70078 (PMC12266749; doi:10.1111/acel.70078)
Supplement: Supplementary file 2 — Tables S1‐S3. [file ACEL-24-e70078-s001.docx]

**Supplemental Tables**

**SQSTM1/p62 orchestrates skin aging via USP7 degradation**

Liu Chen, Xiaoping Wang, Yuchen Wang, Qingxin Yao, Yunyao Liu, Yongcheng Zhu, He Huang, Hedan Yang, Yin Yang, Yuan He, and Lei Qiang

**Table S1.** The sequences of the primers used for RT-PCR analyses, related to STAR Methods.

| Gene | Species | Primer sequence | |
| --- | --- | --- | --- |
| p62 | Human | Forward | GCCCTGAGGAACAGATGGAG |
|  |  | Reverse | AGATGTGGGTACAAGGCAGC |
|  | Mouse | Forward | TGGTGGGAACTCGCTATAAGTG |
|  |  | Reverse | CCAAAGTGTCCATGTTTCAGC |
| TP53 | Human | Forward | CCCAGCCAAAGAAGAAACCA |
|  |  | Reverse | GTTCCAAGGCCTCATTCAGCT |
|  | Mouse | Forward | AGCTCCCATCACTTCATCCC |
|  |  | Reverse | GTGTGGGGTAGGGTGAGATT |
| USP7 | Human | Forward | ATGCAGAGATGGCTGGGAAC |
|  |  | Reverse | CTCAGGGCCACATTCCCATT |
|  | Mouse | Forward | ATCCGATGACTGCTGTGGAA |
|  |  | Reverse | CTGCCACCCAAAGGAAAAGG |
| GAPDH | Human | Forward | AATCCCATCACCATCTTCCA |
|  |  | Reverse | TGGACTCCACGACGTACTCA |
|  | Mouse | Forward | CCTTAAGAGGGATGCTGCC |
|  |  | Reverse | ACTGTGCCGTTGAATTTGCC |
| ATG7 | Human | Forward | TGCCTCACCAGGTTCTTGAT |
|  |  | Reverse | CGCTCATGTCCCAGATCTCA |
| Il-1β | Mouse | Forward | TGTAATGAAAGACGGCACACC |
|  |  | Reverse | TCTTCTTTGGGTATTGCTTGG |
| Il-6 | Mouse | Forward | ACAACCACGGCCTTCCCTAC |
|  |  | Reverse | TCTCATTTCCACGATTTCCCAG |
| Il-8 | Mouse | Forward | GTGTTGGCCCAATTACTAACAG |
|  |  | Reverse | TCCCGAATTGGAAAGGGAAATA |
| Il-10 | Mouse | Forward | GCTCTTACTGACTGGCATGAG |
|  |  | Reverse | CGCAGCTCAGGAGCATGTG |
| Il-12 | Mouse | Forward | TCTTCTCACCGTGCACATCC |
|  |  | Reverse | TGGCCAAACTGAGGTGGTTT |
| Il-17 | Mouse | Forward | TGAGTCCAGGGAGAGCTTCA |
|  |  | Reverse | TTCATTGCGGTGGAGAGTCC |
| TNF-α | Mouse | Forward | GCCTCTTCTCATTCCTGCTT |
|  |  | Reverse | CTCCTCCACTTGGTGGTTTG |

**Table S2.** The sequences of the primers used for plasmid construction, related to STAR Methods.

| Vector | | Gene | Primer sequence | | |  |
| --- | --- | --- | --- | --- | --- | --- |
| pLenti-puro vector | | p62 | Forward | | GTCGACGGGCGTCGCTCACCGTGAAG |  |
|  |  |  | Reverse | | GGATCCCTAGCGGCCGCGCAACG |  |
| PGEX-6P-1 vector | | p62 | Forward | | CCGTCAGATCCGGTACCGCTATGGCGTCGCTCACCGTG |  |
|  |  |  | Reverse | | GTAATCTGGAACATCGTATGGGTAGCGGCCGCGCAAC |  |
|  |  | USP7 | Forward | | CCCTGGGATCCCCGGAATTCATGAACCACCAGCAGCAGC |  |
|  |  |  | Reverse | | CACGATGCGGCCGCTCGAGTCAGTTATGGATTTTAATGGCC |  |
| pCMV-  GFP  vactor | | p62 | Forward | | GATGTGGAGGAGAGTTCCAGCACAGAGGAGAAGAG |  |
|  |  |  | Reverse | | GAACTCTCCTCCACATCGATATCAACTTCAATG |  |
| pCMV-  MYC  vactor | | p62 | Forward | | CGAGCTCAAGCTTCGAATTCGCGTCGCTCACCGTGAAG |  |
|  |  |  | Reverse | | GTTATCTAGATCCGGTGGATCCCTAGCGGCCGCGCAACGGC |  |
|  |  | p62  ΔPB1 | Forward | | CGCGAGATTGAGTGCCGGCGGGACCACCG |  |
|  |  |  | Reverse | | CCGGCACTCAATCTCGCGCGCCGCGTCCTCC |  |
|  |  | p62  ΔZZ | Forward | | CAATGTGATCACCAAGCTCGCATTCCCCAGCC |  |
|  |  |  | Reverse | | GAACTTGGTGATCACATTGGGGTGCACCATG |  |
|  |  | p62  ΔTBS | Forward | | GCAGAATCAGGCATTGAAGTTGATATCGATGG |  |
|  |  |  | Reverse | | CTTCAATGCCTGATTCTGCCGTGGGGCCAGGG |  |
|  |  | p62  ΔLIR | Forward | | GTCCGAGGGGTCAAAAGAAGTGGACCCGTCAC |  |
|  |  |  | Reverse | | CTTCTTTTGACCCCTCGGACTCCAAGGCGATCTTTC |  |
|  |  | p62  ΔKIR | Forward | | GTGGACCCGGAATCCGAAGGGCCAAGCTCTC |  |
|  |  |  | Reverse | | CTTCGGATTCCGGGTCCACTTCTTTTGAAGAC |  |
|  |  | p62  ΔUBA | Forward | | GTACCCACATTAGGGATCCACCGGATCTAGATAAC |  |
|  |  |  | Reverse | | GGATCCCTAATGTGGGTACAAGGCAGCTTCC |  |
|  |  |  | Forward | | CTTCGAGCTCAAGCTTCGAATTCATGAACCACCAGCAGCAG |  |
|  |  |  | Reverse | | CTAGATCCGGTGGATCCTCAGTTATGGATTTTAATGGCC |  |
|  |  | USP7-TCD | Forward | | CTTCGAGCTCAAGCTTCGAATTCATGAACCACCAGCAGCAG |  |
|  |  |  | Reverse | | CTAGATCCGGTGGATCCTCATTCCTGCCGCTCCTTCCGC |  |
|  |  | USP7-TRAF | Forward | | CTTCGAGCTCAAGCTTCGAATTCATGAACCACCAGCAGCAG |  |
|  |  |  | Reverse | | CTAGATCCGGTGGATCCTCACCACGCAACTCCATGGGGAG |  |
|  |  | USP7-Ubl | Forward | | GGAAGCCGATCCCGAGCTGGCTGCTAGTG |  |
|  |  |  | Reverse | | CGGGATCGGCTTCCTGCCGCTCCTTCCGC |  |
| pCMV-  HA  vactor | | USP7 ΔUbl1 | Forward | | GGAAGCCGATCCCGAGCTGGCTGCTAGTG |  |
|  |  |  | Reverse | | CGGGATCGGCTTCCTGCCGCTCCTTCCGC |  |
|  |  | USP7 ΔUbl2 | Forward | | GATAAAGATCATGACCCTGAAAATGATAACAGTG |  |
|  |  |  | Reverse | | CAGGGTCATGATCTTTATCAAACTTGGG |  |
|  |  | USP7 ΔUbl3 | Forward | | CTCTACCACAAGATGAAAATCACAGACTTTG |  |
|  |  |  | Reverse | | CATCTTGTGGTAGAGATCTCGGAAATAC |  |
|  |  | USP7 ΔUbl4 | Forward | | GAACAGGATCCCTTTGGACCAGGTGGAC |  |
|  |  |  | Reverse | | CAAAGGGATCCTGTTCTCAAAGTCTGTG |  |
|  |  | USP7 ΔUbl5 | Forward | | GACAAATGAGGATCCACCGGATCTAG |  |
|  |  |  | Reverse | | GATCCTCATTTGTCTATGTCCACCTGGTC |  |
| pLKO.1 puro | | p62 | | | Forward 1 | CCGGCCTCTGGGCATTGAAGTTGATCTCGAGATCAACTTCAATGCCCAGAGGTTTTT |
|  |  |  |  |  | Reverse 1 | AATTCAAAAACCTCTGGGCATTGAAGTTGATCTCGAGATCAACTTCAATGCCCAGAGG |
|  |  |  |  |  | Forward 2 | CCGGGCAGATGAGAAAGATCGCCTTCTCGAGAAGGCGATCTTTCTCATCTGCTTTTT |
|  |  |  |  |  | Reverse 2 | AATTCAAAAAGCAGATGAGAAAGATCGCCTTCTCGAGAAGGCGATCTTTCTCATCTGC |
|  |  |  |  |  | Forward 3 | CCGGCCGAATCTACATTAAAGAGAACTCGAGTTCTCTTTAATGTAGATTCGGTTTTT |
|  |  |  |  |  | Reverse 3 | AATTCAAAAACCGAATCTACATTAAAGAGAACTCGAGTTCTCTTTAATGTAGATTCGG |
|  |  |  |  |  | Forward 4 | CCGGCGAGGAATTGACAATGGCCATCTCGAGATGGCCATTGTCAATTCCTCGTTTTT |
|  |  |  |  |  | Reverse 4 | AATTCAAAAACGAGGAATTGACAATGGCCATCTCGAGATGGCCATTGTCAATTCCTCG |
|  |  |  |  |  | Forward 5 | CCGGTGCCTAATGGCTTTCACTTTCCTCGAGGAAAGTGAAAGCCATTAGGCATTTTTT |
|  |  |  |  |  | Reverse 5 | AATTCAAAAAATGCCTAATGGCTTTCACTTTCCTCGAGGAAAGTGAAAGCCATTAGGCA |
| pLKO.1 hygro | | p53 | | | Forward 1 | CCGGCGGCGCACAGAGGAAGAGAATCTCGAGATTCTCTTCCTCTGTGCGCCGTTTTT |
|  |  |  |  |  | Reverse 1 | AATTCAAAAACGGCGCACAGAGGAAGAGAATCTCGAGATTCTCTTCCTCTGTGCGCCG |
|  |  |  |  |  | Forward 2 | CCGGTCAGACCTATGGAAACTACTTCTCGAGAAGTAGTTTCCATAGGTCTGATTTTT |
|  |  |  |  |  | Reverse 2 | AATTCAAAAATCAGACCTATGGAAACTACTTCTCGAGAAGTAGTTTCCATAGGTCTGA |
|  |  |  |  |  | Forward 3 | CCGGGTCCAGATGAAGCTCCCAGAACTCGAGTTCTGGGAGCTTCATCTGGACTTTTT |
|  |  |  |  |  | Reverse 3 | AATTCAAAAAGTCCAGATGAAGCTCCCAGAACTCGAGTTCTGGGAGCTTCATCTGGAC |
|  |  |  |  |  | Forward 4 | CCGGGAGGGATGTTTGGGAGATGTACTCGAGTACATCTCCCAAACATCCCTCTTTTT |
|  |  |  |  |  | Reverse 4 | AATTCAAAAAGAGGGATGTTTGGGAGATGTACTCGAGTACATCTCCCAAACATCCCTC |
|  |  |  |  |  | Forward 5 | CCGGCACCATCCACTACAACTACATCTCGAGATGTAGTTGTAGTGGATGGTGTTTTTG |
|  |  |  |  |  | Reverse 5 | AATTCAAAAACACCATCCACTACAACTACATCTCGAGATGTAGTTGTAGTGGATGGTG |
| pLKO.1 hygro | | USP7 | | | Forward 1 | CCGGGTGTCCTATATCCAGTGTAAACTCGAGTTTACACTGGATATAGGACACTTTTT |
|  |  |  |  |  | Reverse 1 | AATTAAAAAGTGTCCTATATCCAGTGTAAATCGAGTTTACACTGGATATAGGACAC |
|  |  |  |  |  | Forward 2 | CCGGCGTGGTGTCAAGGTGTACTAACTCGAGTTAGTACACCTTGACACCACGTTTTT |
|  |  |  |  |  | Reverse 2 | AATTAAAAACGTGGTGTCAAGGTGTACTAACTCGAGTTAGTACACCTTGACACCACG |
|  |  |  |  |  | Forward 3 | CCGGCCTGGATTTGTGGTTACGTTACTCGAGTAACGTAACCACAAATCCAGGTTTTT |
|  |  |  |  |  | Reverse 3 | AATTAAAAACCTGGATTTGTGGTTACGTTACTCGAGTAACGTAACCACAAATCCAGG |
|  |  |  |  |  | Forward 4 | CCGGCCAGCTAAGTATCAAAGGAAACTCGAGTTTCCTTTGATACTTAGCTGGTTTTT |
|  |  |  |  |  | Reverse 4 | AATTAAAAACCAGCTAAGTATCAAAGGAAACTCGAGTTTCCTTTGATACTTAGCTGG |
|  |  |  |  |  | Forward 5 | CCGGTGTATCTATTGACTGCCCTTTCTCGAGAAAGGGCAGTCAATAGATACATTTTT |
|  |  |  |  |  | Reverse 5 | AATTAAAAATGTATCTATTGACTGCCCTTTCTCGAGAAAGGGCAGTCAATAGATACA |
| LentiCRISPR | | p62 | | | Forward 1 | ACGCTACACAAGTCGTAGTC |
|  |  |  |  |  | Reverse 1 | GACTACGACTTGTGTAGCGT |
|  |  |  |  |  | Forward 2 | CGACTTGTGTAGCGTCTGCG |
|  |  |  |  |  | Reverse 2 | CGCAGACGCTACACAAGTCG |

**Table S3.** The sequences of the primers used for plasmid site-directed mutagenesis, related to STAR Methods.

| Gene | Mutation sites |  | Primer sequence |
| --- | --- | --- | --- |
| pCMV-  MYC-p62 | Y67G | Forward | CACGGCCGCGATGAGGACGGGGACTTGGTTGC |
|  |  | Reverse | TCCTCATCGCGGCCGTGCGCCTGGAAGCCGCC |
|  | A76E | Forward | GGTTGAGTTTTCCAGTGACGAGGAATTGACAA |
|  |  | Reverse | CACTGGAAAACTCAACCAAGTCCCCGTCCTCAT |
|  | G405D | Forward | GTCCATGGATTTCTCTGATGAAGGCGGCTGGC |
|  |  | Reverse | CAGAGAAATCCATGGACAGCATCTGGGAGAGG |
|  | S407A | Forward | TGGGCTTCGCCGATGAAGGCGGCTGGCTCACC |
|  |  | Reverse | TGGGCTTCGCCGATGAAGGCGGCTGGCTCACC |
|  | T412R | Forward | TGAAGGCGGCCGCCTCACCAGGCTCCTGCAGAC |
|  |  | Reverse | TGAGGCGGCCGCCTTCATCAGAGAAGCCCATG |
|  | Q418L | Forward | CCTGCTCACCAAGAACTATGACATCGGAGCGGC |
|  |  | Reverse | CTTGGTGAGCAGGAGCCTGGTGAGCCAGCCGCCTTC |
|  | K420T | Forward | GCAGACCACCAACTATGACATCGGAGCGGCTC |
|  |  | Reverse | CATAGTTGGTGGTCTGCAGGAGCCTGGTGAGC |
|  | T430P | Forward | TCTGGACCCAATCCAGTATTCAAAGCATCCCCC |
|  |  | Reverse | ACTGGATGTGAGTCCAGAGCCGCTCCGATGTCA |
|  | Q432A | Forward | CACCATCGCGTATTCAAAGCATCCCCCGCCGT |
|  |  | Reverse | TTGAATACGCGATGGTGTCCAGAGCCGCTCCG |
| pCMV-  HA*-*USP7 | E987A | Forward | GAGAATGCCATGCTTGTCACAGTGGCGCATTTCC |
|  |  | Reverse | GCATGGCATTCTCTTTGTCTATGTCCACCTGGTCC |
|  | M988P | Forward | GAATGAGCCGCTTGTCACAGTGGCGCATTTCCAC |
|  |  | Reverse | GTGACAAGCGGCTCATTCTCTTTGTCTATGTCCAC |
|  | T991S | Forward | CTTGTCAGCGTGGCGCATTTCCACAAAGAGGTCTTC |
|  |  | Reverse | CGCCACGCTGACAAGCATCTCATTCTCTTTGTCTATG |
|  | V992A | Forward | GTCACAGCCGCGCATTTCCACAAAGAGGTCTTC |
|  |  | Reverse | GCGCGGCTGTGACAAGCATCTCATTCTCTTTGTC |
|  | A993G | Forward | CACAGTGGGACATTTCCACAAAGAGGTCTTCGG |
|  |  | Reverse | GGAAATGTCCCACTGTGACAAGCATCTCATTC |
|  | F995G | Forward | GCGCATGGCCACAAAGAGGTCTTCGGAACGTTCGG |
|  |  | Reverse | CTTTGTGGCCATGCGCCACTGTGACAAGCATCTC |
|  | L1028R | Forward | GAGCCTGCGCGACATCCAGGAGAAGGAGTTTGAG |
|  |  | Reverse | GGATGTCGCGCAGGCTCTGGATTCGCTTCATCAC |
